# Supplementary material for: Introducing an integrated maternity care pathway for women with a history of small-for-gestational-age: Evaluation of its effect on care process and clinical outcomes
Source: PLoS One. 2026 Jan 28;21(1):e0341071. doi: 10.1371/journal.pone.0341071 (PMC12851471; doi:10.1371/journal.pone.0341071)
Supplement: Attachment — Submitted filename: PONE-D-25-11450_R1.pdf [file pone.0341071.s004.pdf]

PLOS ONE

Introducing an integrated maternity care pathway for women with a history of small-for-gestational-age: evaluation of its effect on care process and clinical outcomes

--Manuscript Draft--

**Manuscript Number:**	PONE-D-25-11450R1
**Article Type:**	Research Article
**Full Title:**	Introducing an integrated maternity care pathway for women with a history of small-for-gestational-age: evaluation of its effect on care process and clinical outcomes
**Short Title:**	Evaluation of an integrated maternity care pathway for women with a history of SGA
**Corresponding Author:**	Anne Hermans, M.D.<br>Erasmus Medical Centre: Erasmus MC<br>Rotterdam, NETHERLANDS, KINGDOM OF THE
**Keywords:**	Integrated maternity care<br>small-for-gestational-age in obstetric history<br>care proces<br>perinatal outcomes
**Abstract:**	Introduction<br>This study focusses on the implementation of an integrated cae pathway for women with SGA in their obstetric history that pursues value-based healthcare. This study aims to 1) Determine whether the integrated care pathway led to a reduction in the number of antenatal secondary care consultations, as as indicator of care efficacy, and 2) compare clinical outcomes for women with a history of SGA before and after implemenetation of the integrated care pathway.<br><br>Methods<br><br>Retrospective cohort study including data from pregnant women with a history of SGA within integrated maternity care organisation Annature, 2017-2020. Intervention was an integrated care pathway (2018). Pre- and post-intervention periods were compared assessing prenatal secondary care visits, place and mode of delivery, and perinatal outcomes.<br><br>Results<br><br>The implementation of the care pathway for pregnant women with a history of SGA led to a reduction in mean numbe of prenatal secondary care consultations per pregnancy from 11 in 2017 to 5 in 2020, and fewer inductions of labour (78 (34.2%) vs 127 (26.8%), p=0.045). Additionally, the number of births in primary care increased (35 (15.4%) vs 136 (28.8%), p<0.001) with no significant adverse impact on neonatal outcomes in the post-intervention period compared to the pre-intervention period.<br><br>Conclusion<br><br>The implementation of the care pathway for pregnant women with a history of SGA resulted in a reduction in prenatal secondary care consultations and fewer inductions of labour. Additionally, the number of births in primary care increased, with no significant adverse impact on neonatal outcomes in the post-intervention period compared to the pre-intervention period.
**Order of Authors:**	A C.M. Hermans, M.D.
	J. Spaan
	M.A. Hermus
	A.M. Hietkamp
	J. Visser

Powered by Editorial Manager® and ProduXion Manager® from Aries Systems Corporation

	A. Franx, Prof, M.D., PhD
	J. van der Kooy
**Response to Reviewers:**	Please also see the attached file ´Response to Reviewers´ in which the respond to specific reviewer and editor comments are more clearly stated:<br><br>Comments to the Author<br><br>1. Is the manuscript technically sound, and do the data support the conclusions?<br><br>The manuscript must describe a technically sound piece of scientific research with data that supports the conclusions. Experiments must have been conducted rigorously, with appropriate controls, replication, and sample sizes. The conclusions must be drawn appropriately based on the data presented.<br>Reviewer #1: Yes<br>Reviewer #2: Yes<br>____<br>2. Has the statistical analysis been performed appropriately and rigorously?<br>Reviewer #1: Yes<br>Reviewer #2: Yes<br>____<br>3. Have the authors made all data underlying the findings in their manuscript fully available?<br><br>The PLOS Data policy requires authors to make all data underlying the findings described in their manuscript fully available without restriction, with rare exception (please refer to the Data Availability Statement in the manuscript PDF file). The data should be provided as part of the manuscript or its supporting information, or deposited to a public repository. For example, in addition to summary statistics, the data points behind means, medians and variance measures should be available. If there are restrictions on publicly sharing data—e.g. participant privacy or use of data from a third party—those must be specified.<br>Reviewer #1: No<br>Reviewer #2: Yes<br>We confirm that our submission contains all raw data required to replicate the results of our study. All relevant data, including the data required to replicate all study findings, as well as related metadata and methods), are provided within the manuscript and its Supporting Information files.<br>The data were pseudo anonymised prior to use, in accordance with institutional protocols and national ethical standards. The data were provided by the regional hospital and the Netherlands Perinatal Registry. One of the co-authors, who serves as the project leader, was involved in the development and preparation of the database. As the study involved retrospective analysis of pseudo anonymised medical records, it was exempt from institutional ethical review, in line with national regulations as outlined by the Central Committee on Research Involving Human Subjects (CCMO) in the Netherlands (https://english.ccmo.nl). We further confirm that the dataset is complete and that there are no restrictions on data sharing. All data relevant to the findings reported in the manuscript are available. The pseudo anonymised dataset can be made available upon reasonable request to the corresponding author, in full accordance with the journal's data sharing policy. Should the Editor or reviewers require any further information or clarification, we would be more than happy to provide additional details as needed.<br><br>____<br>4. Is the manuscript presented in an intelligible fashion and written in standard English?<br><br>PLOS ONE does not copyedit accepted manuscripts, so the language in submitted articles must be clear, correct, and unambiguous. Any typographical or grammatical errors should be corrected at revision, so please note any specific errors here.<br>Reviewer #1: Yes<br>Reviewer #2: Yes<br>____<br>5. Review Comments to the Author

Please use the space provided to explain your answers to the questions above. You may also include additional comments for the author, including concerns about dual publication, research ethics, or publication ethics. (Please upload your review as an attachment if it exceeds 20,000 characters)

Reviewer #1: There is an overlap of pre-intervention and post-intervention time. As per the paper "The SGA care pathway was initially developed in December 2017, with clinical implementation occurring gradually between January and May 2018. In the pre intervention group, included women who gave birth between January 2017 and September
99 2018. In the post-intervention group, we included women who gave birth from 2018 until December
100 2020." I dont understand the reason for this overlap and why couldnt it be all together avoided?

As of January 2018, women with a history of SGA were enrolled in the newly developed integrated SGA care pathway at the intake of a subsequent pregnancy. Prior to this, care for these women had not yet been formally structured within a standardized integrated care protocol. Consequently, the pre-intervention period was defined as women who delivered between January 2017 and September 2018, while the post-intervention period comprised women who delivered between October 2018 and December 2020. In the post-intervention group, we included women who gave birth from October 2018 until December 2020. Consequently, there is no overlap between the two groups. We added this explanation to the method section:

Methods section, lines 97-104

As of January 2018, women with a history of SGA were enrolled in the newly developed integrated SGA care pathway at the intake of a subsequent pregnancy. Prior to this, care for these women had not yet been formally structured within a standardized integrated care protocol. Consequently, the pre-intervention period was defined as women who delivered between January 2017 and September 2018, while the post-intervention period comprised women who delivered between October 2018 and December 2020. In the post-intervention group, we included women who gave birth from October 2018 until December 2020.

One of the aim of the study was to see a reduction in the number of in-hospital visits? Does it mean inpatient visits?? thats quite confusing

We thank the reviewer for this observation and would like to clarify that we indeed assessed secondary care consultations as a measure of care efficacy.(5) By secondary care, we refer to consultations carried out by either an obstetrician or a clinical midwife in the hospital outpatient clinic.

To provide context for international readers, we have described the Dutch maternity care system and recent regional developments in care integration. The Netherlands employs a tiered maternity care system based on estimated risk for adverse outcomes: primary care for low-risk pregnancies is provided by community midwives; hospital-based obstetricians manage intermediate-risk pregnancies in secondary care; and university medical centres provide tertiary care for high-risk pregnancies. Approximately 80% of pregnant women receive prenatal care across these tiers, but referrals from primary to secondary care can disrupt continuity, risking loss of essential medical information. Poor integration between tiers may lead to redundant procedures, such as unnecessary ultrasound investigations, potentially resulting in over-medicalisation.

Our underlying hypothesis is that more integrated care, with improved cross-tier collaboration, will reduce unnecessary duplication of services and avoid non-essential consultations. Within an integrated care pathway, responsibilities and tasks of primary and secondary care providers are more clearly aligned, which facilitates efficient care allocation, prevents overlap, and promotes optimal use of healthcare resources.

Reviewer #2: Subject: REVIEWERS' COMMENT ON MANUSCRIPT NUMBER: PONE-D-25-11450

Title: Introducing an integrated maternity care pathway for women with a history of small-for-gestational-age: evaluation of its effect on care process and clinical outcomes

I would like to congratulate the authors on the manuscript submitted for review and

publication, which focuses on an important aspect of maternal and perinatal care, focused on task shifting with optimal maternal-fetal outcome.
The article, the implementation of an integrated maternity care pathway for women with a history of small-for-gestational-age (SGA) pregnancies, evaluated the care pre and postdelivery, reducing the focus of care on the secondary health facility, except if required, to a integrated maternity care pathway, with deliveries within the primary level of care, assessing efficacy of the pathway by reduced antenatal in-hospital visits and labor inductions, and compared clinical outcomes before and after its implementation.
The outcomes were: a reduction in antenatal visits, fewer labour inductions, an increase in Primary Care births with standard monitoring of Neonatal Outcomes, reduced caesarean deliveries and improved neonatal outcomes.
The research article satisfies the following criteria:
1. The study presents the results of original research. Yes, it does.
2. Results reported have not been published elsewhere.
3. The experiments/ methods, statistics, and other analyses are performed to a high technical standard and are described in sufficient detail.
4. Conclusions are presented in an appropriate fashion and are supported by the data. Yes, this has been presented in an appropriate fashion, and it is supported by the data.
5. The article is presented in an intelligible fashion with good flow and is written in standard English.
6. The research meets all applicable standards for the ethics of experimentation and research integrity. Yes, they did not require ethical clearance, but they ensured deidentified data was used and ensured institutional protocol and national guidelines.
7. The article adheres to appropriate reporting guidelines and community standards for data availability. Yes, this was done, and a statement on how to access data was included.

I recommend that this article be published as it meets high standards and has researched an area of importance in the management of women and their fetuses. It has provided insights into the implementation of an integrated care pathway in clinical practice, focusing on pregnant women with a history of SGA.

Thank you for the opportunity to review this manuscript for publication

We are grateful to the reviewer for their thoughtful feedback and the considerable time and effort they invested in evaluating our manuscript.

____

6. PLOS authors have the option to publish the peer review history of their article (what does this mean?). If published, this will include your full peer review and any attached files.

If you choose “no”, your identity will remain anonymous but your review may still be made public.

Do you want your identity to be public for this peer review? For information about this choice, including consent withdrawal, please see our Privacy Policy.
Reviewer #1: Yes: zaheena shamsul islam
Reviewer #2: No

____

[NOTE: If reviewer comments were submitted as an attachment file, they will be attached to this email and accessible via the submission site. Please log into your account, locate the manuscript record, and check for the action link "View Attachments". If this link does not appear, there are no attachment files.]

While revising your submission, please upload your figure files to the Preflight Analysis and Conversion Engine (PACE) digital diagnostic tool, https://pacev2.apexcovantage.com/. PACE helps ensure that figures meet PLOS requirements. To use PACE, you must first register as a user. Registration is free. Then, login and navigate to the UPLOAD tab, where you will find detailed instructions on how to use the tool. If you encounter any issues or have any questions when using

	PACE, please email PLOS at figures@plos.org. Please note that Supporting Information files do not need this step.<br><br><br>References<br>1. Leitich H, Egarter C, Husslein P, Kaider A, Schemper M. A meta-analysis of low dose aspirin for the prevention of intrauterine growth retardation. Br J Obstet Gynaecol [Internet]. 1997 [cited 2025 Aug 14];104(4):450–9. Available from: https://pubmed.ncbi.nlm.nih.gov/9141582/<br>2. Bujold E, Roberge S, Lacasse Y, Bureau M, Audibert F, Marcoux S, et al. Prevention of preeclampsia and intrauterine growth restriction with aspirin started in early pregnancy: A meta-analysis. Obstet Gynecol [Internet]. 2010 [cited 2025 Aug 14];116(2):402–14. Available from: https://pubmed.ncbi.nlm.nih.gov/20664402/<br>3. NVOG-richtlijn Foetale groeirestrictie (FGR).<br>4. Roberge S, Odibo AO, Bujold E. Aspirin for the Prevention of Preeclampsia and Intrauterine Growth Restriction. Clin Lab Med. 2016 Jun;36(2):319–29.<br>5. Peahl AF, Gourevitch RA, Luo EM, Fryer KE, Moniz MH, Dalton VK, et al. Right-Sizing Prenatal Care to Meet Patients' Needs and Improve Maternity Care Value. Obstet Gynecol. 2020;135(5):1027–37.
**Additional Information:**	
**Question**	**Response**
**Financial Disclosure**<br><br>Enter a financial disclosure statement that describes the sources of funding for the work included in this submission. Review the submission guidelines for detailed requirements. View published research articles from *PLOS ONE* for specific examples.<br><br>This statement is required for submission and **will appear in the published article** if the submission is accepted. Please make sure it is accurate.<br><br>**Funded studies**<br>Enter a statement with the following details:<br>• Initials of the authors who received each award<br>• Grant numbers awarded to each author<br>• The full name of each funder<br>• URL of each funder website<br>• Did the sponsors or funders play any role in the study design, data collection and analysis, decision to publish, or preparation of the manuscript?<br><br>Did you receive funding for this work?	Yes
Please add funding details.	ZonMw 10100022020013. The funders had no role in study design, data collection and

as follow-up to "**Financial Disclosure**<br><br>Enter a financial disclosure statement that describes the sources of funding for the work included in this submission. Review the submission guidelines for detailed requirements. View published research articles from *PLOS ONE* for specific examples.<br><br>This statement is required for submission and **will appear in the published article** if the submission is accepted. Please make sure it is accurate.<br><br>**Funded studies**<br>Enter a statement with the following details:<br>• Initials of the authors who received each award<br>• Grant numbers awarded to each author<br>• The full name of each funder<br>• URL of each funder website<br>• Did the sponsors or funders play any role in the study design, data collection and analysis, decision to publish, or preparation of the manuscript?<br><br>Did you receive funding for this work?"	analysis, decision to publish, or preparation of the manuscript.
Please select the country of your main research funder (please select carefully as in some cases this is used in fee calculation).<br>as follow-up to "**Financial Disclosure**<br><br>Enter a financial disclosure statement that describes the sources of funding for the work included in this submission. Review the submission guidelines for detailed requirements. View published research articles from *PLOS ONE* for specific examples.<br><br>This statement is required for submission and **will appear in the published article** if the submission is accepted. Please make sure it is accurate.	NETHERLANDS - NL

**Funded studies**<br>Enter a statement with the following details:<br>• Initials of the authors who received each award<br>• Grant numbers awarded to each author<br>• The full name of each funder<br>• URL of each funder website<br>• Did the sponsors or funders play any role in the study design, data collection and analysis, decision to publish, or preparation of the manuscript?<br><br>Did you receive funding for this work?"	
**Competing Interests**<br><br>Use the instructions below to enter a competing interest statement for this submission. On behalf of all authors, disclose any competing interests that could be perceived to bias this work—acknowledging all financial support and any other relevant financial or non-financial competing interests.<br><br>This statement is required for submission and **will appear in the published article** if the submission is accepted. Please make sure it is accurate and that any funding sources listed in your Funding Information later in the submission form are also declared in your Financial Disclosure statement.<br><br>View published research articles from *PLOS ONE* for specific examples.	The authors have declared that no competing interests exist.

Powered by Editorial Manager® and ProduXion Manager® from Aries Systems Corporation

**NO authors have competing interests**<br><br>Enter: *The authors have declared that no competing interests exist.*<br><br>**Authors with competing interests**<br><br>Enter competing interest details beginning with this statement:<br><br>*I have read the journal's policy and the authors of this manuscript have the following competing interests: [insert competing interests here]*<br><br>* typeset	
**Ethics Statement**<br><br>Enter an ethics statement for this submission. This statement is required if the study involved:<br><br>• Human participants<br>• Human specimens or tissue<br>• Vertebrate animals or cephalopods<br>• Vertebrate embryos or tissues<br>• Field research<br><br>Write "N/A" if the submission does not require an ethics statement.<br><br>General guidance is provided below. Consult the submission guidelines for detailed instructions. **Make sure that all information entered here is included in the Methods section of the manuscript.**	N/A

**Format for specific study types**<br><br>**Human Subject Research (involving human participants and/or tissue)**<br>• Give the name of the institutional review board or ethics committee that approved the study<br>• Include the approval number and/or a statement indicating approval of this research<br>• Indicate the form of consent obtained (written/oral) or the reason that consent was not obtained (e.g. the data were analyzed anonymously)<br><br>**Animal Research (involving vertebrate animals, embryos or tissues)**<br>• Provide the name of the Institutional Animal Care and Use Committee (IACUC) or other relevant ethics board that reviewed the study protocol, and indicate whether they approved this research or granted a formal waiver of ethical approval<br>• Include an approval number if one was obtained<br>• If the study involved *non-human primates*, add *additional details* about animal welfare and steps taken to ameliorate suffering<br>• If anesthesia, euthanasia, or any kind of animal sacrifice is part of the study, include briefly which substances and/or methods were applied<br><br>**Field Research**<br><br>Include the following details if this study involves the collection of plant, animal, or other materials from a natural setting:<br>• Field permit number<br>• Name of the institution or relevant body that granted permission	
**Data Availability**<br><br>Authors are required to make all data underlying the findings described fully available, without restriction, and from the time of publication. PLOS allows rare exceptions to address legal and ethical concerns. See the PLOS Data Policy and FAQ for detailed information.	Yes - all data are fully available without restriction

A Data Availability Statement describing where the data can be found is required at submission. Your answers to this question constitute the Data Availability Statement and **will be published in the article**, if accepted.<br><br>**Important:** Stating 'data available on request from the author' is not sufficient. If your data are only available upon request, select 'No' for the first question and explain your exceptional situation in the text box.<br><br>Do the authors confirm that all data underlying the findings described in their manuscript are fully available without restriction?	
**Describe where the data may be found in full sentences. If you are copying our sample text, replace any instances of XXX with the appropriate details.**<br><br>• If the data are **held or will be held in a public repository**, include URLs, accession numbers or DOIs. If this information will only be available after acceptance, indicate this by ticking the box below. For example: *All XXX files are available from the XXX database (accession number(s) XXX, XXX.).*<br>• If the data are all contained **within the manuscript and/or Supporting Information files**, enter the following: *All relevant data are within the manuscript and its Supporting Information files.*<br>• If neither of these applies but you are able to provide **details of access elsewhere**, with or without limitations, please do so. For example:<br><br>*Data cannot be shared publicly because of [XXX]. Data are available from the XXX Institutional Data Access / Ethics Committee (contact via XXX) for researchers who meet the criteria for access to confidential data.*<br><br>*The data underlying the results presented in the study are available from (include the name of the third party*	All relevant data are within the manuscript and its Supporting Information files.

and contact information or URL).<br>• This text is appropriate if the data are owned by a third party and authors do not have permission to share the data.<br><br>* typeset	
Additional data availability information:	

Powered by Editorial Manager® and ProduXion Manager® from Aries Systems Corporation

Manuscript
Click here to access/download;Manuscript;250920 SGA care pathway manuscript_clean_1.docx

Introducing an integrated maternity care pathway for women with a history of small-for-gestational-age: evaluation of its effect on care process and clinical outcomes

A.C.M. Hermans*[1], J. Spaan[2], M.A. Hermus[3], A.M. Hietkamp[2], J.Visser[2], A. Franx[1], J. van der Kooy[1]

[1] Department of Obstetrics and Gynaecology, Erasmus Medical University Centre, Rotterdam, the Netherlands.

[2] Department of Obstetrics and Gynaecology, Amphia hospital, Breda, the Netherlands

[3] Midwifery Practice Oosterhout, Oosterhout, the Netherlands

*Corresponding author

E-mail: a.c.m.hermans@erasmusmc.nl

Abstract

Introduction This study focusses on the implementation of an integrated care pathway for women with SGA in their obstetric history that pursues value-based healthcare. This study aims to 1) Determine whether the integrated care pathway led to a reduction in the number of antenatal secondary care consultations, as an indicator of care efficacy, and 2) compare clinical outcomes for women with a history of SGA before and after implementation of the integrated care pathway.

Methods Retrospective cohort study including data from pregnant women with a history of SGA within integrated maternity care organisation Annature, 2017-2020. Intervention was an integrated care pathway (2018). Pre- and post-intervention periods were compared assessing prenatal secondary care consultations, place and mode of delivery, and perinatal outcomes.

Results The implementation of the care pathway for pregnant women with a history of SGA led to a reduction in mean number of prenatal secondary care consultations per pregnancy from 11 in 2017 to 5 in 2020, and fewer inductions of labour (78 (34.2%) vs 127 (26.8%), p=0.045). Additionally, the number of births in primary care increased (35 (15.4%) vs 136 (28.8%), p<0.001) with no significant adverse impact on neonatal outcomes in the post-intervention period compared to the pre-intervention period.

Conclusion The implementation of the care pathway for pregnant women with a history of SGA resulted in a reduction in prenatal secondary care consultations and fewer inductions of labour. Additionally, the number of births in primary care increased, with no significant adverse impact on neonatal outcomes in the post-intervention period compared to the pre-intervention period.

Introduction

Despite various initiatives aimed at improving the organization and enhancing inter professional collaboration within maternity care in the Netherlands, prompted by reports of relatively high perinatal mortality rates compared to other European countries (1), the perinatal mortality rate has remained stagnant since 2016 (2–4). Preterm birth and low birth weight, specifically below the 10th percentile, commonly referred to as small-for-gestational-age (SGA), are the most frequent antecedents of perinatal mortality (5–8). A key challenge in addressing this issue is organisational improvement through the implementation of a maternity care model that pursues value-based healthcare. In striving toward a patient-centred integrated maternity care system, it is crucial to strike a balance that prevents situations in which care provided 'too much, too soon' or 'too little, too late' (9)

The Netherlands employs a tiered maternity care system based on estimated risk for adverse outcomes. Primary care for low-risk pregnancies is provided by community midwives, hospital-based obstetricians manage intermediate-risk pregnancies in secondary care, and university medical centres provide tertiary care for high-risk pregnancies. Approximately 80% of pregnant women receive prenatal care across these different tiers. However, referrals from primary to secondary care can disrupt continuity, increasing the risk of losing critical medical information. Inadequate integration between these levels of care may result in duplicated procedures—such as unnecessary ultrasound examinations—thereby contributing to over-medicalisation. Therefore, The Dutch Steering Group Pregnancy and Childbirth, installed by the Ministery of Health as a response to the performance in the EURO-PERISTAT benchmark in 2008, emphasized the need for an integrated maternity care model to enhance interprofessional collaboration between primary and secondary care. (1)

The integrated maternity care approach entails joint responsibility between community midwives and secondary obstetric care providers in hospital, replacing previous practices in which such intermediate-risk pregnancies were exclusively managed in secondary care. Integrated maternity care aims to provide structured, patient-centred prenatal care: Providing

primary midwife-led care close to home whenever possible and secondary obstetrician-led care in hospitals when necessary.

Previous research indicates that an integrated approach to maternity care may positively influence perinatal outcomes, such as improved infant birth weight (10). Furthermore, evidence shows that midwife-led continuity models are associated with higher maternal satisfaction while achieving perinatal outcomes comparable to other care models (11), along with potential cost-saving benefits.(9,12)

In the Southwestern region, these findings eventually led to the foundation of an integrated maternity care organisation (IMCO). Within the IMCO, obstetric professionals from primary and secondary care work within an integrated patient record. (13) Furthermore, they collaboratively developed standardized protocols and pathways to enhance patient-centred care. These care pathways outline antenatal consultations and clearly set the roles of community midwives, clinical midwives, and obstetricians. Within this model, intermediate-risk pregnancies, such as those involving a history of small-for-gestational-age (SGA) infants, are managed through integrated care, with close interprofessional collaboration between primary and secondary care providers.

So far, limited studies have evaluated the effect of maternity care incorporating integrated care pathways on the pre- and perinatal care processes and clinical outcomes, particularly for women with intermediate- or high-risk pregnancies.(14,15). This study contributes to existing literature by evaluating an integrated care pathway characterized by extensive interprofessional collaboration. The first aim of our study was to determine whether the integrated care pathway led to a reduction in the number of antenatal secondary care consultations, as an indicator of care efficacy. The secondary objective was to compare clinical outcomes for women with a history of SGA before and after the implementation of the integrated care pathway. Lastly, we compared perinatal outcomes between the pre- and post-intervention periods, stratified by whether a woman delivered an SGA neonate or a neonate with normal birth weight.

Methods

Retrospective cohort study in which the SGA care pathway was assessed before and after its implementation. We included data from women with singleton pregnancies with a history of SGA receiving care from IMCO Annature. As of January 2018, women with a history of SGA were enrolled in the newly developed integrated SGA care pathway at the intake of a subsequent pregnancy. Prior to this, care for these women had not yet been formally structured within a standardized integrated care protocol. Consequently, the pre-intervention period was defined as women who delivered between January 2017 and September 2018, while the post-intervention period comprised women who delivered between October 2018 and December 2020. In the post-intervention group, we included women who gave birth from October 2018 until December 2020. Multiple pregnancies (n=6) were excluded. SGA was defined as a birth weight below the 10th percentile, which was calculated using the Hoftiezer curve (16). The birth weight percentile was automatically calculated upon registration of the birth weight, and this method remained consistent throughout the study period. The file review using patient records took place from January 8, 2022, to March 24, 2022.

SGA care pathway: what it entails

Before the implementation of the SGA care pathway, there was no structured protocol for managing intermediate-risk pregnancies, resulting in considerable practice variation within the IMCO. The introduction of the integrated care pathway established a more systematic approach by clearly defining the responsibilities of primary and secondary care providers and organising multidisciplinary antenatal consultations at specific time intervals.

As shown in Fig 1, the SGA care pathway includes antenatal counselling on the use of low-dose aspirin, in accordance with the Dutch Society of Obstetrics and Gynaecology (NVOG) guideline on fetal growth restriction. In addition, the pathway incorporates prenatal

ultrasounds at 27, 31, and 35 weeks of gestation to monitor foetal growth. Foetal growth restriction was defined as an estimated foetal weight (EFW) or abdominal circumference (AC) below the 10th percentile, or a growth deviation of ≥20 percentiles within two weeks. If no signs of foetal growth restriction were detected at the 35-week visit and no other complications were present, further management of pregnancy and labour proceeded in primary care.

Outcomes

For the evaluation of the SGA care pathway, two comparisons were performed: 1) care and perinatal outcomes were compared between the period before (January 2017 to September 2018) and after (October 2018 to December 2020) the introduction of the integrated care pathway for women with previous SGA; and (2) perinatal outcomes were compared between the pre- and post-intervention periods, stratified by whether a woman delivered an SGA neonate (the "SGA group") or a neonate with normal birthweight (the "non-SGA group").

Baseline characteristics were compared between the pre- and post-intervention periods, and included maternal age, pre-pregnancy BMI, ethnicity, living in a deprived neighbourhood (defined as a residential area with various infrastructural and social shortcomings), and smoking (at pregnancy intake).

Care factors included: mean number of antenatal consultations per pregnancy per year in primary care or secondary care for the years 2017 to 2020. Antenatal consultations in primary care were available for analysis from 2018 onward as they were registered within the integrated patient record. Therefore, we have chosen to calculate the mean number of antenatal consultations per year instead of making a pre- post-intervention comparison.

Furthermore, we evaluated the place and mode of delivery, distinguishing between the location where labour began and the eventual place of delivery (i.e., primary care or secondary care). Additionally, perinatal outcomes included the prevalence of meconium-

stained amniotic fluid, an Apgar score below 7 after 5 minutes, an umbilical artery pH below 7.05, paediatric involvement, and perinatal mortality, defined as the sum of stillbirths and early neonatal deaths within the first 7 days, which is expressed per 1,000 live births.(17) Paediatric involvement was defined as any involvement of the paediatrician, which included both paediatric consultations as neonatal admissions to the ward or the NICU.

Statistical analysis

The analyses were conducted using IBM SPSS Statistics version 25. The Chi-square test was used to compare baseline characteristics per year. For numbers <5, Fisher's exact test was applied. The threshold for statistical significance was set at alpha <0.05. All data for this study were derived from the Netherlands Perinatal Registry and from the electronic patient records of the regional hospital in the Breda region, the Netherlands. The data were anonymised prior to access for research purposes, in accordance with institutional protocols and applicable privacy regulations. The anonymisation process was carried out before the start of the analysis and was not performed by the study analysts themselves. The project leader, who is one of the co-authors, was involved in the development of the study database but did not have access to identifying information during the analysis phase.
Access to identifiable patient information was restricted to authorised healthcare professionals within the Department of Obstetrics and Gynaecology, who had such access only as part of their clinical responsibilities. The research team only received anonymised data for analysis. The retrospective use of anonymised medical records was exempt from institutional review by the Medical Ethics Committee, in compliance with Dutch national guidelines.

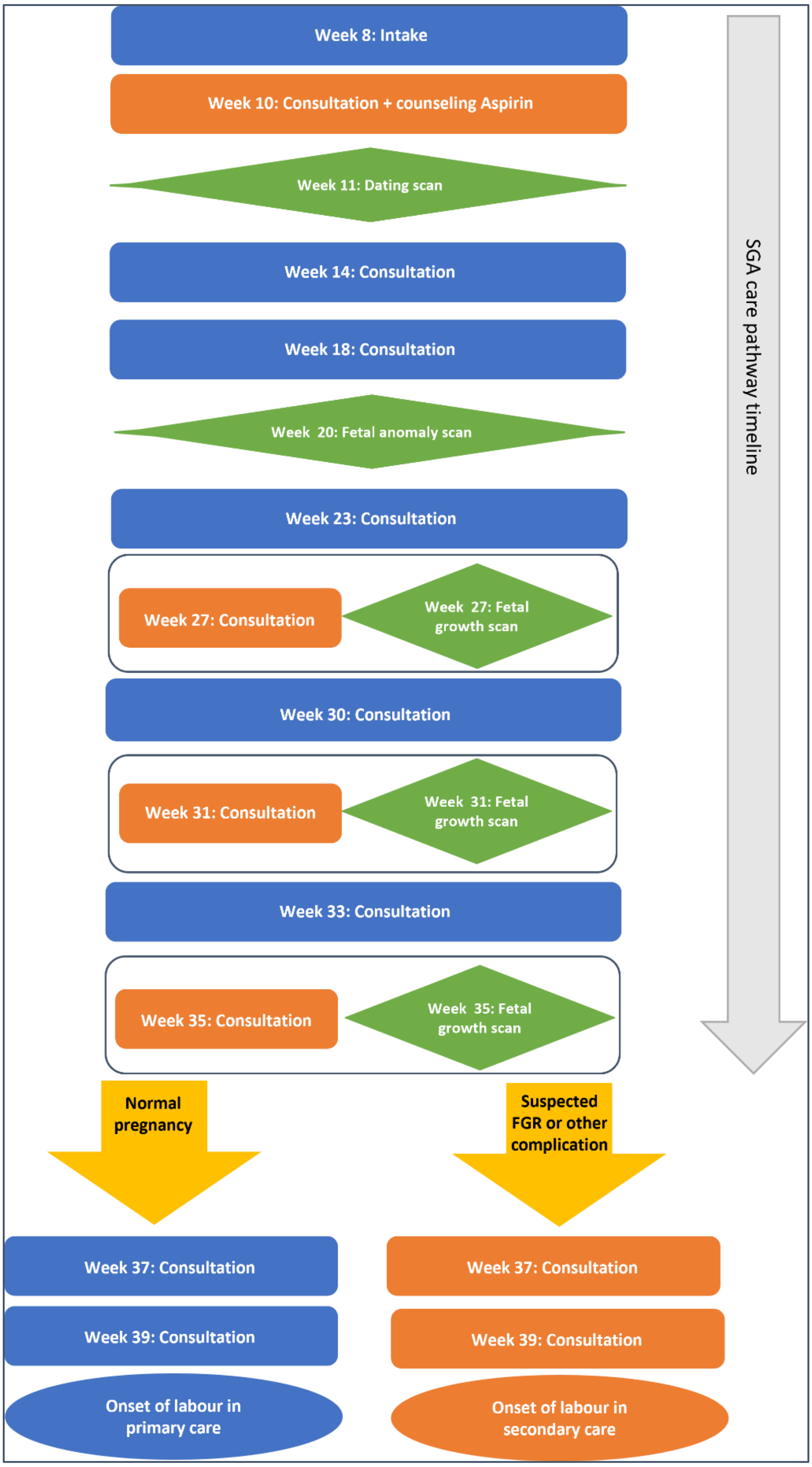

Fig 1. Visualised SGA care pathway in which the trajectory of prenatal care is displayed in the post-intervention period. Blue: Antenatal visit in primary care; orange: Antenatal visit in secondary care; green: prenatal ultrasound; yellow arrows: at 35 weeks of gestation the risk for foetal growth restriction (FGR) are assessed. When no foetal growth retardation or any other complication of pregnancy was detected at this antenatal visit, further follow-up of pregnancy and childbirth could take place in a primary care setting.

Results

Demographic characteristics

Between January 2017 and December 2020, 701 pregnant women with a history of SGA received care from IMCO Annature. Of these women, 228 received care before, and 473 received care after introduction of the SGA care pathway. Table 1 demonstrates the distribution of demographic characteristics of the ‘before’ and ‘after’ groups, with a significant decline in hypertensive disorders of pregnancy of 19 (8.3%) in the pre-intervention period compared to 15 (3.2%) in the post-intervention period (p=0.003). No significant differences were observed in maternal age, pre-pregnancy BMI, ethnicity, living in a deprived neighbourhood or smoking.

Table 1. Baseline characteristics of pregnant women with small-for-gestational-age in their obstetric history. Pre-intervention period: January 2017 until September 2018; post-intervention period: October 2018 until December 2020. Data in n (%). Maternal age in years, BMI: body mass index (kg/ m^2), low-dose Ascal: 80mg per day per os. [x] Fisher’s exact test. *Alpha<0.05.

Demographic characteristics	Pre-intervention period (n=228)	Post-intervention period (n=473)	P-value
**Maternal age (years)**			0.895
≤ 25	6 (2.6)	14 (3.0)	
25-34	150 (65.8)	303 (64.1)	
≥ 35	72 (31.6)	156 (33.0)	
**Pre-pregnancy BMI (kg/m^2)**			0.159
<25	130 (57.0)	291 (61.5)	
25-29.99	60 (26.3)	122 (25.8)	
30-34.99	24 (10.5)	47 (9.9)	
≥ 35	14 (6.1)	13 (2.7)	
**Ethnicity**			0.066
Dutch	154 (67.5)	351 (74.2)	
Non-Dutch	74 (32.50	122 (25.8)	
**Deprived neighbourhood**	30 (13.2)	48 (10.1)	0.235
**Smoking**	38 (17.2)	39 (12.6)	0.137
**Hypertensive disorders of pregnancy**	19 (8.3)	15 (3.2)	0.003
**Diabetes Mellitus**	1 (0.4)	1 (0.2)	0.597

**Gestational Diabetes Mellitus**	22 (9.6)	50 (10.6)	0.706

Antenatal consultations

The number of antenatal consultations is demonstrated in Table 2. The number of consultations in primary care is only available for analysis in the period 2018-2020 and remained stable over the years. The number of antenatal consultations in secondary care declined from 11 in 2017 to 7 in 2018-2019, and to 5 in 2020.

Table 2. Mean number of antenatal care consultations in primary care and secondary care per pregnancy for women with small-for-gestational-age in their obstetric history within integrated care organisation Annature, Breda region, the Netherlands.

	2017 (n=99)	2018 (n=156)	2019 (n=212)	2020 (n=222)
**Mean number of primary care consultations per pregnancy per year**	-	8	8	8
**Mean number of secondary care consultations per pregnancy per year**	11	7	7	5

Place and mode of delivery

Table 3 shows that following the introduction of the SGA care pathway, 279 women (59.0%) used low-dose aspirin, compared to 102 women (44.7%) prior to its introduction ($p<0.001$). Additionally, significantly more women started labour in primary care after the pathway was introduced (214 women, 45.2%, versus 65 women, 28.5%, $p<0.001$). Moreover, a significantly higher number of women gave birth in primary care (136 women, 28.8%, versus 35 women, 15.4%; $p<0.001$). Furthermore, labour induction was performed in 127 women (26.8%) after the introduction of the SGA care pathway, compared to 78 women (34.2%) in the pre-intervention period ($p=0.045$). There was a trend towards lower caesarean section rates from 44 (19.3%) towards 65 (13.7%, $p=0.057$).

Perinatal outcomes

Furthermore, table 3 shows that the neonatal outcomes did not differ significantly in the pre- and post-intervention groups.

Table 3. Mode of delivery and neonatal outcomes for pregnant women with small-for-gestational-age (SGA) in their obstetric history. Pre-intervention period: January 2017 until September 2018 (n=228) versus post-intervention period: October 2018 until December 2020 (n=473). Data are in n (%). *Alpha<0.05. [x] Fisher's exact test.

	Pre-intervention period (n=228)	Post-intervention period (n=473)	P-value
**Obstetric characteristics**			
**Maternal low-dose Aspirin use**	102 (44.7)	279 (59.0)	**<0.001***
**Place of delivery**			
**Start of labour in primary care**<br>- **SGA-infant**	65 (28.5)<br>12/65 (18.5)	214 (45.2)<br>42/214 (19.6)	**<0.001***<br>0.835
**Start of labour in secondary care**<br>- **SGA-infant**	163 (71.5)<br>56/163 (34.4)	259 (54.8)<br>81/259 (31.3)	**<0.001***<br>0.510
**Induction of labour**	78 (34.2)	127 (26.8)	**0.045***
**Place of birth in primary care**	35 (15.4)	136 (28.8)	**<0.001***
**Place of birth in secondary care**	193 (84.6)	337 (71.2)	**<0.001***
**Mode of delivery**			
**Vaginal delivery**	178 (78.1)	397 (83.9)	0.153
**Vacuum assisted delivery[x]**	6 (2.6)	11 (2.3)	0.121
**Caesarean section**<br>- **Primary caesarean section**<br>- **Secondary caesarean section**	44 (19.3)<br>28 (12.3)<br>16 (7.0)	65 (13.7)<br>44 (9.5)<br>21 (4.4)	0.057<br>0.261<br>0.153
**Neonatal outcomes**			
**SGA**	68 (29.8)	123 (26.0)	0.287
**Meconium-containing amniotic fluid**	31 (13.6)	63 (13.3)	0.920
**Apgar score <7 (5 minutes post-partum)[x]**	3 (1.3)	8 (1.7)	1.000
**Ph <7.05 (5 minutes post-partum)[x]**	2 (0.9)	5 (1.1)	1.000
**Paediatrician involvement**	123 (53.9)	225 (47.6)	0.114
**Perinatal mortality[x]**	1 (0.4)	1 (0.2)	0.545

Comparing perinatal outcomes stratified for SGA group versus non-SGA group

Table 4 presents a comparison of perinatal outcomes between the pre- and post-intervention periods, stratified by whether a woman delivered an SGA neonate (the “SGA group”) or a neonate with normal birth weight (the “non-SGA group”). Table 4 shows that in the SGA-group the paediatrician was less frequently involved postpartum after the introduction of the SGA care pathway (pre: 63 (92.6%) versus post: 101 (82.1%), p=0.045). In the non-SGA group, there were fewer caesarean sections after the introduction of the integrated care pathway (pre: 34 (21.3%) versus post: 46 (13.1%) p=0.019).

Table 4. Mode of delivery and neonatal outcomes for pregnant women with small-for-gestational-age (SGA) in their obstetric history; SGA (n=191) versus non-SGA (n=510), divided into the following groups: Pre-intervention period: January 2017 until September 2018 versus post-intervention period: October 2018 until December 2020. Data are in n (%). NA: not available. [x] Fisher’s exact test. *Alpha<0.05

	SGA (n=191)			Non-SGA (n=510)		
Mode of delivery	Pre-intervention period (n=68)	Post-intervention period (n=123)	p-value	Pre-intervention period (n=160)	Post-Intervention period (n=350)	p-value
**Induction of labour**	30 (44.1)	39 (31.7)	0.087	48 (30.0)	88 (25.1)	0.250
**Vaginal delivery**	56 (82.4)	102 (82.9)	0.827	122 (76.3)	295 (84.3)	0.065
**Vacuum assisted delivery[x]**	2 (2.9)	2 (1.6)	0.617 [x]	4 (2.5)	9 (2.6)	1.000
**Caesarean section**	10 (14.7)	19 (15.4)	0.891	34 (21.3)	46 (13.1)	**0.019***
- **Primary caesarean section[x]**	5 (7.4)	12 (10.6)	0.608	23 (14.4)	32 (9.1)	0.077
- **Secondary caesarean section[x]**	5 (7.4)	7 (5.7)	0.757	11 (6.9)	14 (4.0)	0.163
Neonatal outcomes						
**Meconium-containing amniotic fluid**	10 (14.7)	15 (12.2)	0.622	21 (13.1)	48 (13.7)	0.857
**Apgar score <7 (5 minutes post-partum) [x]**	0 (0.0)	3 (2.4)	0.265	3 (1.9)	5 (1.4)	0.710
**Ph <7.05 (5 minutes post-partum) [x]**	0 (0.0)	2 (1.6)	0.539	2 (1.3)	3 (0.9)	0.651
**Paediatrician involvement**	63 (92.6)	101 (82.1)	**0.045***	60 (37.5)	124 (35.4)	0.651
**Perinatal mortality[x]**	0 (0.0)	0 (0.0)	NA	1 (0.6)	1 (0.3)	0.529

Discussion

The implementation of the care pathway for pregnant women with a history of SGA led to a reduction in prenatal secondary care consultations, and fewer labour inductions. Additionally, the number of births in primary care increased, with no significant adverse impact on neonatal outcomes in the post-intervention period compared to the pre-intervention period.

Our finding regarding the decline in mean number of antenatal secondary care consultations might be interpreted as an indicator of improved efficacy of care (18). Furthermore, previous literature found that reduced antenatal consultations are associated with fewer interventions and improvement of perinatal outcomes in low-risk pregnancies (19). Another randomized controlled trial in a tertiary academic centre found that a reduced frequency prenatal care model with fewer onsite appointments supplemented with virtual visit was associated with higher patient satisfaction without compromising perinatal outcomes (20)

After implementation of the care pathway, there were fewer inductions of labour (IOL) compared to the pre-intervention period. Unfortunately, the reason for IOL were not available for analysis within our study. This finding is not in line with previous research that found no association between model of maternity care and IOL rate (12)

Our study demonstrates that following the implementation of the SGA care pathway, more women gave birth in a primary care setting with similar perinatal outcomes. This shift toward primary care is significant, as it allowed more women with intermediate-risk pregnancies to deliver in primary care compared to the period before the integrated care pathway was implemented. Providing care in a primary care setting has been shown to positively impact patient experience and perinatal outcomes. (12). Similarly, shifting care to primary maternity care settings has proven feasible for women at risk of preterm birth.(20)

Regarding our secondary objectives, stratified by whether a woman delivered an SGA neonate or a neonate with normal birthweight, we found that following implementation of the

SGA care pathway, the number of caesarean sections decreased in women who, in their current pregnancy, gave birth to an infant with a normal birthweight (referred to as non-SGA group). We hypothesize the integrated care pathway with a clear structure probably has facilitated this decline, although proof of causality is difficult with this 'before-after' study design. The integrated patient record enabled enhanced peer consultation between primary and secondary care providers. Together with the structured care pathway, this led to improved informational continuity of care and patient-centred care, leveraging knowledge of prior events and individual circumstances. (21,22) This finding aligns with a systematic review showing that organisational interventions, such as team midwifery models, are associated with reduced caesarean section rates.(23)

We observed a 10% decrease in paediatric involvement in the SGA group, to 82.1% following implementation of the integrated SGA care pathway. Assessment of all 101 cases separately revealed that paediatric involvement was often omitted after uncomplicated home births with a birthweight just below the 10th percentile. In these cases, feeding advice was provided by the attending community midwife, and hospital transfer was deemed unnecessary. No adverse neonatal outcomes, such as neonatal admissions, were reported in these cases. This suggests that post-homebirth hospital transfers for glucose monitoring may pose a barrier in certain situations.

Strengths and limitations

A key strength of this study is its provision of insights into the implementation of an integrated care pathway in clinical practice, encompassing all pregnant women in the region. From a value-based healthcare perspective, we evaluated this integrated care pathway to identify areas for future improvement. However, its retrospective design is a limitation, and we were not able to include long-term neonatal outcomes. Finally, the concurrent COVID-19 pandemic may have contributed to the observed reduction in antenatal consultations in secondary care in the end of 2020.

Future research should focus on a comprehensive cost-effectiveness analysis to provide robust evidence regarding the implementation of integrated maternity care. While this study has assessed the integrated care pathway from a value-based healthcare perspective and assessed the care process, which provide insights into care efficacy, a more comprehensive economic evaluation is required to draw more robust conclusions.

Conclusion

The implementation of the care pathway for pregnant women with a history of SGA resulted in a reduction in antenatal secondary care consultations and fewer inductions of labour. Additionally, the number of births in primary care increased, with no significant adverse impact on neonatal outcomes in the post-intervention period compared to the pre-intervention period.

Financial Disclosure Statement

ZonMw 10100022020013. The funders had no role in study design, data collection and analysis, decision to publish, or preparation of the manuscript.

Ethics statement

The data used in this study were provided by the regional hospital, and the Netherlands Perinatal Registry , and the project leader of the study, who is one of the co-authors, was involved in the development of the database. The anonymisation of the data was performed prior to analysis and in accordance with institutional protocols. The retrospective use of anonymized medical records exempted institutional review of the Medical Ethics Committee. This complies with national guidelines (https://english.ccmo.nl/).

Acknowledgements

The Netherlands Perinatal Registry, Utrecht, the Netherlands, kindly provided permission for the data analyses included.

References

1. Stuurgroep zwangerschap en geboorte. Veilige zorg rond zwangerschap en geboorte. 2009;1–86.
2. Kerncijfers middelengebruik van vrouwen rond de zwangerschap in 2021.
3. Broeders L, Achterberg PW, Waelput AJM, Ravelli ACJ, Kwee A, Groenendaal F, et al. [Decrease in foetal and neonatal mortality in the Netherlands; comparison with other Euro-Peristat countries in 2004, 2010 and 2015]. Ned Tijdschr Geneeskd. 2019 Jul;163.
4. EUROPEAN PERINATAL HEALTH REPORT. [cited 2022 Jul 14]; Available from: www.europeristat.com
5. Bonsel GJ (Gouke), Birnie E (Erwin), Denktaş S (Semiha), Steegers EAP (Eric), Poeran VJJ (Jashvant). Lijnen in de Perinatale Sterfte [Internet]. 2010. Available from: http://www.narcis.nl/publication/RecordID/oai:repub.eur.nl:23454
6. Katz J, Lee ACC, Kozuki N, Lawn JE, Cousens S, Blencowe H, et al. Mortality risk in preterm and small-for-gestational-age infants in low-income and middle-income countries: a pooled country analysis. Lancet (London, England) [Internet]. 2013 [cited 2023 Feb 22];382(9890):417–25. Available from: https://pubmed.ncbi.nlm.nih.gov/23746775/
7. Lee ACC, Katz J, Blencowe H, Cousens S, Kozuki N, Vogel JP, et al. National and regional estimates of term and preterm babies born small for gestational age in 138 low-income and middle-income countries in 2010. Lancet Glob Heal [Internet]. 2013 Sep [cited 2023 Feb 22];1(1). Available from: https://pubmed.ncbi.nlm.nih.gov/25103583/
8. Pulver LS, Guest-Warnick G, Stoddard GJ, Byington CL, Young PC. Weight for gestational age affects the mortality of late preterm infants. Pediatrics [Internet]. 2009

Jun [cited 2023 Feb 22];123(6). Available from: https://pubmed.ncbi.nlm.nih.gov/19482740/

9. Martin E, Ayoub B, Miller YD. A systematic review of the cost-effectiveness of maternity models of care. BMC Pregnancy Childbirth [Internet]. 2023 Dec 1 [cited 2025 Jan 4];23(1):859. Available from: https://pmc.ncbi.nlm.nih.gov/articles/PMC10717830/

10. Kroll-Desrosiers AR, Crawford SL, Moore Simas TA, Rosen AK, Mattocks KM. Improving Pregnancy Outcomes through Maternity Care Coordination: A Systematic Review. Women's Heal Issues [Internet]. 2016;26(1):87–99. Available from: http://dx.doi.org/10.1016/j.whi.2015.10.003

11. Sandall J, Fernandez Turienzo C, Devane D, Soltani H, Gillespie P, Gates S, et al. Midwife continuity of care models versus other models of care for childbearing women. Cochrane Database Syst Rev. 2024 Apr 10;2024(4).

12. Chapman S. Midwife-led continuity models versus other models of care for childbearing women, Sandall et al (2015). Vol. 19, Practising Midwife. 2016. 34–35 p.

13. Hermans A, Spaan J, Hermus M, Visser J, Research Collaboration A, Franx A, et al. Implementation of integrated maternity care in the southwestern region of the Netherlands: evaluation of its effect on preterm birth, low birthweight infants and number of secondary care consultations. BMJ Open [Internet]. 2024 [cited 2024 Oct 29];14:69556. Available from: http://bmjopen.bmj.com/

14. Donnellan-Fernandez RE, Creedy DK, Callander EJ. Cost-effectiveness of continuity of midwifery care for women with complex pregnancy: A structured review of the literature. Health Econ Rev. 2018;8(1).

15. Yeshitila YG, Gold L, Abimanyi-Ochom J, Riggs E, Daba TT, Le HND. Effectiveness and cost-effectiveness of models of maternity care for women from migrant and

refugee backgrounds in high-income countries: A systematic review. Soc Sci Med. 2024 Oct 1;358:117250.

16. Hoftiezer L, Hof MHP, Dijs-Elsinga J, Hogeveen M, Hukkelhoven CWPM, van Lingen RA. From population reference to national standard: new and improved birthweight charts. Am J Obstet Gynecol [Internet]. 2019 Apr 1 [cited 2023 Feb 22];220(4):383.e1-383.e17. Available from: http://www.ajog.org/article/S0002937818322336/fulltext

17. Perinatale sterfte | CBS [Internet]. [cited 2025 Jan 23]. Available from: https://www.cbs.nl/nl-nl/onze-diensten/methoden/begrippen/perinatale-sterfte

18. Peahl AF, Gourevitch RA, Luo EM, Fryer KE, Moniz MH, Dalton VK, et al. Right-Sizing Prenatal Care to Meet Patients' Needs and Improve Maternity Care Value. Obstet Gynecol. 2020;135(5):1027–37.

19. Carter EB, Tuuli MG, Caughey AB, Odibo AO, Macones GA, Cahill AG. Number of prenatal visits and pregnancy outcomes in low-risk women. J Perinatol. 2016 Mar 1;36(3):178–81.

20. Butler Tobah YS, LeBlanc A, Branda ME, Inselman JW, Morris MA, Ridgeway JL, et al. Randomized comparison of a reduced-visit prenatal care model enhanced with remote monitoring. Am J Obstet Gynecol [Internet]. 2019 Dec 1 [cited 2025 Jan 27];221(6):638.e1-638.e8. Available from: http://www.ajog.org/article/S0002937819308105/fulltext

21. Haggerty JL, Reid RJ, Freeman GK, Starfield BH, Adair CE, McKendry R. Continuity of care: a multidisciplinary review. BMJ Br Med J [Internet]. 2003 Nov 11 [cited 2023 Feb 28];327(7425):1219. Available from: /pmc/articles/PMC274066/

22. Vos JFJ, Boonstra A, Kooistra A, Seelen M, Van Offenbeek M. The influence of electronic health record use on collaboration among medical specialties. BMC Health Serv Res. 2020;20(1):1–11.

23. Chapman A, Nagle C, Bick D, Lindberg R, Kent B, Calache J, et al. Maternity service organisational interventions that aim to reduce caesarean section: a systematic review and meta-analyses. [cited 2023 Nov 5]; Available from: https://doi.org/10.1186/s12884-019-2351-2

Click here to access/download
Supporting Information
250925_anonimised_dataset_PLOS_One.xlsx

Introducing an integrated maternity care pathway for women with a history of small-for-gestational-age: evaluation of its effect on care process and clinical outcomes

A.C.M. Hermans*[1], J. Spaan[2], M.A. Hermus[3], A.M. Hietkamp[2], J.Visser[2], A. Franx[1], J. van der Kooy[1]

[1] Department of Obstetrics and Gynaecology, Erasmus Medical University Centre, Rotterdam, the Netherlands.

[2] Department of Obstetrics and Gynaecology, Amphia hospital, Breda, the Netherlands

[3] Midwifery Practice Oosterhout, Oosterhout, the Netherlands

*Corresponding author

E-mail: a.c.m.hermans@erasmusmc.nl

Abstract

Introduction This study focusses on the implementation of an integrated care pathway for women with SGA in their obstetric history that pursues value-based healthcare. This study aims to 1) Determine whether the integrated care pathway led to a reduction in the number of antenatal secondary care consultations, as an indicator of care efficacy, and 2) compare clinical outcomes for women with a history of SGA before and after implementation of the integrated care pathway.

Methods Retrospective cohort study including data from pregnant women with a history of SGA within integrated maternity care organisation Annature, 2017-2020. Intervention was an integrated care pathway (2018). Pre- and post-intervention periods were compared assessing prenatal secondary care consultations, place and mode of delivery, and perinatal outcomes.

Results The implementation of the care pathway for pregnant women with a history of SGA led to a reduction in mean number of prenatal secondary care consultations per pregnancy from 11 in 2017 to 5 in 2020, and fewer inductions of labour (78 (34.2%) vs 127 (26.8%), p=0.045). Additionally, the number of births in primary care increased (35 (15.4%) vs 136 (28.8%), p<0.001) with no significant adverse impact on neonatal outcomes in the post-intervention period compared to the pre-intervention period.

Conclusion The implementation of the care pathway for pregnant women with a history of SGA resulted in a reduction in prenatal secondary care consultations and fewer inductions of labour. Additionally, the number of births in primary care increased, with no significant adverse impact on neonatal outcomes in the post-intervention period compared to the pre-intervention period.

Introduction

Despite various initiatives aimed at improving the organization and enhancing inter professional collaboration within maternity care in the Netherlands, prompted by reports of relatively high perinatal mortality rates compared to other European countries (1), the perinatal mortality rate has remained stagnant since 2016 (2–4). Preterm birth and low birth weight, specifically below the 10th percentile, commonly referred to as small-for-gestational-age (SGA), are the most frequent antecedents of perinatal mortality (5–8). A key challenge in addressing this issue is organisational improvement through the implementation of a maternity care model that pursues value-based healthcare. In striving toward a patient-centred integrated maternity care system, it is crucial to strike a balance that prevents situations in which care provided ‘too much, too soon’ or ‘too little, too late’ (9)

The Netherlands employs a tiered maternity care system based on estimated risk for adverse outcomes. Primary care for low-risk pregnancies is provided by community midwives, hospital-based obstetricians manage intermediate-risk pregnancies in secondary care, and university medical centres provide tertiary care for high-risk pregnancies. Approximately 80% of pregnant women receive prenatal care across these different tiers. However, referrals from primary to secondary care can disrupt continuity, increasing the risk of losing critical medical information. Inadequate integration between these levels of care may result in duplicated procedures—such as unnecessary ultrasound examinations—thereby contributing to over-medicalisation. Therefore, The Dutch Steering Group Pregnancy and Childbirth, installed by the Ministery of Health as a response to the performance in the EURO-PERISTAT benchmark in 2008, emphasized the need for an integrated maternity care model to enhance interprofessional collaboration between primary and secondary care. (1)

The integrated maternity care approach entails joint responsibility between community midwives and secondary obstetric care providers in hospital, replacing previous practices in which such intermediate-risk pregnancies were exclusively managed in secondary care. Integrated maternity care aims to provide structured, patient-centred prenatal care: Providing

primary midwife-led care close to home whenever possible and secondary obstetrician-led care in hospitals when necessary.

Previous research indicates that an integrated approach to maternity care may positively influence perinatal outcomes, such as improved infant birth weight (10). Furthermore, evidence shows that midwife-led continuity models are associated with higher maternal satisfaction while achieving perinatal outcomes comparable to other care models (11), along with potential cost-saving benefits.(9,12)

In the Southwestern region, these findings eventually led to the foundation of an integrated maternity care organisation (IMCO). Within the IMCO, obstetric professionals from primary and secondary care work within an integrated patient record. (13) Furthermore, they collaboratively developed standardized protocols and pathways to enhance patient-centred care. These care pathways outline antenatal consultations and clearly set the roles of community midwives, clinical midwives, and obstetricians. Within this model, intermediate-risk pregnancies, such as those involving a history of small-for-gestational-age (SGA) infants, are managed through integrated care, with close interprofessional collaboration between primary and secondary care providers.

So far, limited studies have evaluated the effect of maternity care incorporating integrated care pathways on the pre- and perinatal care processes and clinical outcomes, particularly for women with intermediate- or high-risk pregnancies.(14,15). This study contributes to existing literature by evaluating an integrated care pathway characterized by extensive interprofessional collaboration. The first aim of our study was to determine whether the integrated care pathway led to a reduction in the number of antenatal secondary care consultations, as an indicator of care efficacy. The secondary objective was to compare clinical outcomes for women with a history of SGA before and after the implementation of the integrated care pathway. Lastly, we compared perinatal outcomes between the pre- and post-intervention periods, stratified by whether a woman delivered an SGA neonate or a neonate with normal birth weight.

Methods

Retrospective cohort study in which the SGA care pathway was assessed before and after its implementation. We included data from women with singleton pregnancies with a history of SGA receiving care from IMCO Annature. As of January 2018, women with a history of SGA were enrolled in the newly developed integrated SGA care pathway at the intake of a subsequent pregnancy. Prior to this, care for these women had not yet been formally structured within a standardized integrated care protocol. Consequently, the pre-intervention period was defined as women who delivered between January 2017 and September 2018, while the post-intervention period comprised women who delivered between October 2018 and December 2020. In the post-intervention group, we included women who gave birth from October 2018 until December 2020. Multiple pregnancies (n=6) were excluded. SGA was defined as a birth weight below the 10th percentile, which was calculated using the Hoftiezer curve (16). The birth weight percentile was automatically calculated upon registration of the birth weight, and this method remained consistent throughout the study period. The file review using patient records took place from January 8, 2022, to March 24, 2022.

SGA care pathway: what it entails

Before the implementation of the SGA care pathway, there was no structured protocol for managing intermediate-risk pregnancies, resulting in considerable practice variation within the IMCO. The introduction of the integrated care pathway established a more systematic approach by clearly defining the responsibilities of primary and secondary care providers and organising multidisciplinary antenatal consultations at specific time intervals.

~~As shown in Fig 1, the SGA care pathway includes antenatal counselling on the use of low-dose aspirin, in accordance with the Dutch Society of Obstetrics and Gynaecology (NVOG) guideline on fetal growth restriction. In addition, the pathway incorporates prenatal~~

~~ultrasounds at 27, 31, and 35 weeks of gestation to monitor foetal growth.~~ As shown in Fig 1, the SGA care pathway includes antenatal counselling on the use of low-dose aspirin, in accordance with the Dutch Society of Obstetrics and Gynaecology (NVOG) guideline on fetal growth restriction. In addition, the pathway incorporates prenatal ultrasounds at 27, 31, and 35 weeks of gestation to monitor foetal growth. Foetal growth restriction was defined as an estimated foetal weight (EFW) or abdominal circumference (AC) below the 10th percentile, or a growth deviation of ≥20 percentiles within two weeks. If no signs of foetal growth restriction were detected at the 35-week visit and no other complications were present, further management of pregnancy and labour proceeded in primary care.

Outcomes

For the evaluation of the SGA care pathway, two comparisons were performed: 1) care and perinatal outcomes were compared between the period before (January 2017 to September 2018) and after (October 2018 to December 2020) the introduction of the integrated care pathway for women with previous SGA; and (2) perinatal outcomes were compared between the pre- and post-intervention periods, stratified by whether a woman delivered an SGA neonate (the “SGA group”) or a neonate with normal birthweight (the “non-SGA group”).

Baseline characteristics were compared between the pre- and post-intervention periods, and included maternal age, pre-pregnancy BMI, ethnicity, living in a deprived neighbourhood (defined as a residential area with various infrastructural and social shortcomings), and smoking (at pregnancy intake).

Care factors included: mean number of antenatal consultations per pregnancy per year in primary care or secondary care for the years 2017 to 2020. Antenatal consultations in primary care were available for analysis from 2018 onward as they were registered within the integrated patient record. Therefore, we have chosen to calculate the mean number of antenatal consultations per year instead of making a pre- post-intervention comparison.

Furthermore, we evaluated the place and mode of delivery, distinguishing between the location where labour began and the eventual place of delivery (i.e., primary care or secondary care). Additionally, perinatal outcomes included the prevalence of meconium-stained amniotic fluid, an Apgar score below 7 after 5 minutes, an umbilical artery pH below 7.05, paediatric involvement, and perinatal mortality, defined as the sum of stillbirths and early neonatal deaths within the first 7 days, which is expressed per 1,000 live births.(17) Paediatric involvement was defined as any involvement of the paediatrician, which included both paediatric consultations as neonatal admissions to the ward or the NICU.

Statistical analysis

The analyses were conducted using IBM SPSS Statistics version 25. The Chi-square test was used to compare baseline characteristics per year. For numbers <5, Fisher's exact test was applied. The threshold for statistical significance was set at alpha <0.05. All data for this study were derived from the Netherlands Perinatal Registry and from the electronic patient records of the regional hospital in the Breda region, the Netherlands. The data were anonymised prior to access for research purposes, in accordance with institutional protocols and applicable privacy regulations. The anonymisation process was carried out before the start of the analysis and was not performed by the study analysts themselves. The project leader, who is one of the co-authors, was involved in the development of the study database but did not have access to identifying information during the analysis phase.
Access to identifiable patient information was restricted to authorised healthcare professionals within the Department of Obstetrics and Gynaecology, who had such access only as part of their clinical responsibilities. The research team only received anonymised data for analysis. The retrospective use of anonymised medical records was exempt from institutional review by the Medical Ethics Committee, in compliance with Dutch national guidelines.

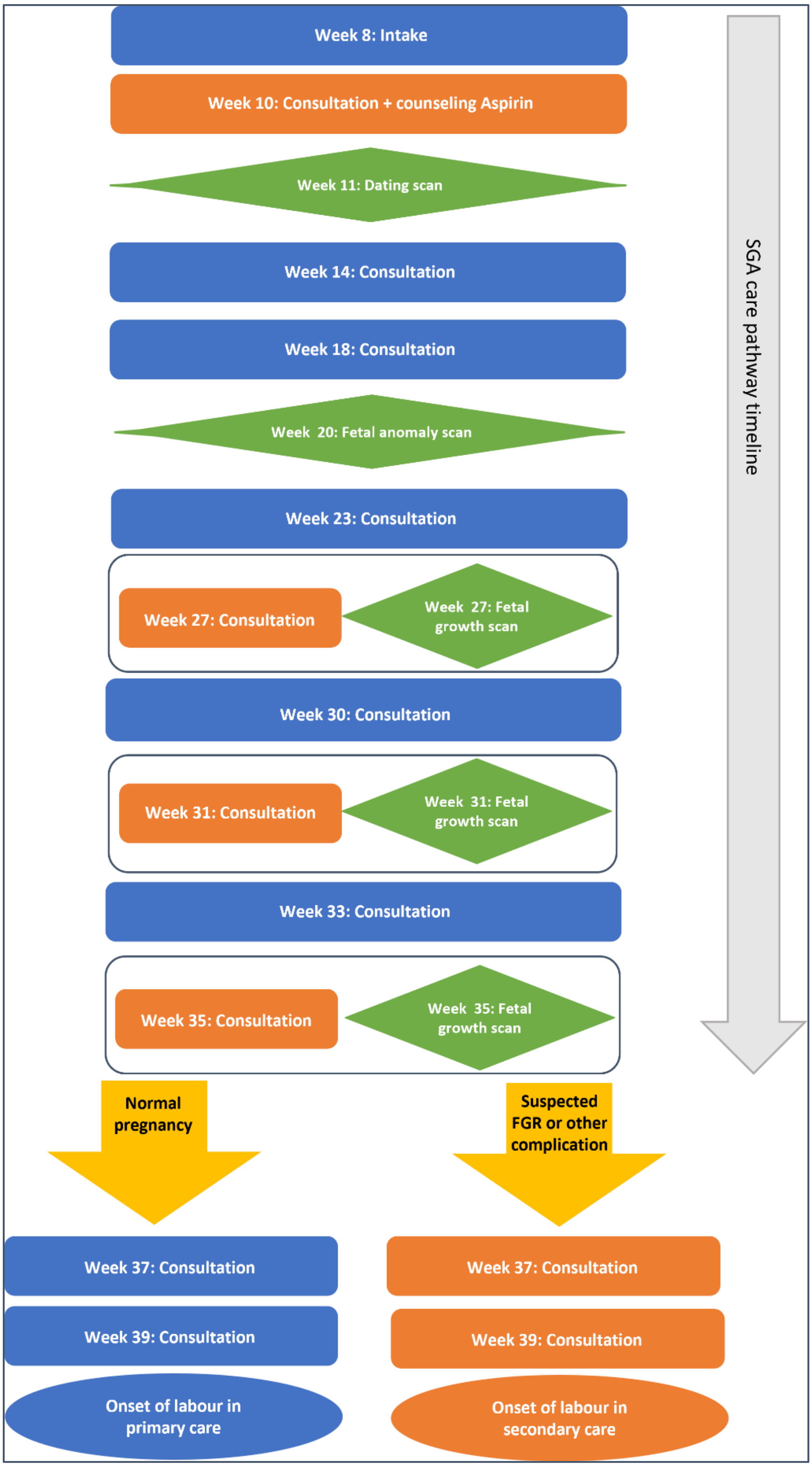

Fig 1. Visualised SGA care pathway in which the trajectory of prenatal care is displayed in the post-intervention period. Blue: Antenatal visit in primary care; orange: Antenatal visit in secondary care; green: prenatal ultrasound; yellow arrows: at 35 weeks of gestation the risk for foetal growth restriction (FGR) are assessed. When no foetal growth retardation or any other complication of pregnancy was detected at this antenatal visit, further follow-up of pregnancy and childbirth could take place in a primary care setting.

Results

Demographic characteristics

Between January 2017 and December 2020, 701 pregnant women with a history of SGA received care from IMCO Annature. Of these women, 228 received care before, and 473 received care after introduction of the SGA care pathway. Table 1 demonstrates the distribution of demographic characteristics of the 'before' and 'after' groups, with a significant decline in hypertensive disorders of pregnancy of 19 (8.3%) in the pre-intervention period compared to 15 (3.2%) in the post-intervention period (p=0.003). No significant differences were observed in maternal age, pre-pregnancy BMI, ethnicity, living in a deprived neighbourhood or smoking.

Table 1. Baseline characteristics of pregnant women with small-for-gestational-age in their obstetric history. Pre-intervention period: January 2017 until September 2018; post-intervention period: October 2018 until December 2020. Data in n (%). Maternal age in years, BMI: body mass index (kg/ m^2), low-dose Ascal: 80mg per day per os. [x] Fisher's exact test. *Alpha<0.05.

Demographic characteristics	Pre-intervention period (n=228)	Post-intervention period (n=473)	P-value
**Maternal age (years)**			0.895
≤ 25	6 (2.6)	14 (3.0)	
25-34	150 (65.8)	303 (64.1)	
≥ 35	72 (31.6)	156 (33.0)	
**Pre-pregnancy BMI (kg/m^2)**			0.159
<25	130 (57.0)	291 (61.5)	
25-29.99	60 (26.3)	122 (25.8)	
30-34.99	24 (10.5)	47 (9.9)	
≥ 35	14 (6.1)	13 (2.7)	
**Ethnicity**			0.066
Dutch	154 (67.5)	351 (74.2)	
Non-Dutch	74 (32.50	122 (25.8)	
**Deprived neighbourhood**	30 (13.2)	48 (10.1)	0.235
**Smoking**	38 (17.2)	39 (12.6)	0.137
**Hypertensive disorders of pregnancy**	19 (8.3)	15 (3.2)	0.003
**Diabetes Mellitus**	1 (0.4)	1 (0.2)	0.597

**Gestational Diabetes Mellitus**	22 (9.6)	50 (10.6)	0.706

Antenatal consultations

The number of antenatal consultations is demonstrated in Table 2. The number of consultations in primary care is only available for analysis in the period 2018-2020 and remained stable over the years. The number of antenatal consultations in secondary care declined from 11 in 2017 to 7 in 2018-2019, and to 5 in 2020.

Table 2. Mean number of antenatal care consultations in primary care and secondary care per pregnancy for women with small-for-gestational-age in their obstetric history within integrated care organisation Annature, Breda region, the Netherlands.

	2017 (n=99)	2018 (n=156)	2019 (n=212)	2020 (n=222)
**Mean number of primary care consultations per pregnancy per year**	-	8	8	8
**Mean number of secondary care consultations per pregnancy per year**	11	7	7	5

Place and mode of delivery

Table 3 shows that following the introduction of the SGA care pathway, 279 women (59.0%) used low-dose aspirin, compared to 102 women (44.7%) prior to its introduction ($p<0.001$). Additionally, significantly more women started labour in primary care after the pathway was introduced (214 women, 45.2%, versus 65 women, 28.5%, $p<0.001$). Moreover, a significantly higher number of women gave birth in primary care (136 women, 28.8%, versus 35 women, 15.4%; $p<0.001$). Furthermore, labour induction was performed in 127 women (26.8%) after the introduction of the SGA care pathway, compared to 78 women (34.2%) in the pre-intervention period ($p=0.045$). There was a trend towards lower caesarean section rates from 44 (19.3%) towards 65 (13.7%, $p=0.057$).

Perinatal outcomes

Furthermore, table 3 shows that the neonatal outcomes did not differ significantly in the pre- and post-intervention groups.

Table 3. Mode of delivery and neonatal outcomes for pregnant women with small-for-gestational-age (SGA) in their obstetric history. Pre-intervention period: January 2017 until September 2018 (n=228) versus post-intervention period: October 2018 until December 2020 (n=473). Data are in n (%). *Alpha<0.05. [x] Fisher's exact test.

	Pre-intervention period (n=228)	Post-intervention period (n=473)	P-value
Obstetric characteristics			
Maternal low-dose Aspirin use	102 (44.7)	279 (59.0)	**<0.001***
Place of delivery			
Start of labour in primary care<br>- SGA-infant	65 (28.5)<br>12/65 (18.5)	214 (45.2)<br>42/214 (19.6)	**<0.001***<br>0.835
Start of labour in secondary care<br>- SGA-infant	163 (71.5)<br>56/163 (34.4)	259 (54.8)<br>81/259 (31.3)	**<0.001***<br>0.510
Induction of labour	78 (34.2)	127 (26.8)	**0.045***
Place of birth in primary care	35 (15.4)	136 (28.8)	**<0.001***
Place of birth in secondary care	193 (84.6)	337 (71.2)	**<0.001***
Mode of delivery			
Vaginal delivery	178 (78.1)	397 (83.9)	0.153
Vacuum assisted delivery[x]	6 (2.6)	11 (2.3)	0.121
Caesarean section<br>- Primary caesarean section<br>- Secondary caesarean section	44 (19.3)<br>28 (12.3)<br>16 (7.0)	65 (13.7)<br>44 (9.5)<br>21 (4.4)	0.057<br>0.261<br>0.153
Neonatal outcomes			
SGA	68 (29.8)	123 (26.0)	0.287
Meconium-containing amniotic fluid	31 (13.6)	63 (13.3)	0.920
Apgar score <7 (5 minutes post-partum)[x]	3 (1.3)	8 (1.7)	1.000
Ph <7.05 (5 minutes post-partum)[x]	2 (0.9)	5 (1.1)	1.000
Paediatrician involvement	123 (53.9)	225 (47.6)	0.114
Perinatal mortality[x]	1 (0.4)	1 (0.2)	0.545

Comparing perinatal outcomes stratified for SGA group versus non-SGA group

Table 4 presents a comparison of perinatal outcomes between the pre- and post-intervention periods, stratified by whether a woman delivered an SGA neonate (the “SGA group”) or a neonate with normal birth weight (the “non-SGA group”). Table 4 shows that in the SGA-group the paediatrician was less frequently involved postpartum after the introduction of the SGA care pathway (pre: 63 (92.6%) versus post: 101 (82.1%), p=0.045). In the non-SGA group, there were fewer caesarean sections after the introduction of the integrated care pathway (pre: 34 (21.3%) versus post: 46 (13.1%) p=0.019).

Table 4. Mode of delivery and neonatal outcomes for pregnant women with small-for-gestational-age (SGA) in their obstetric history; SGA (n=191) versus non-SGA (n=510), divided into the following groups: Pre-intervention period: January 2017 until September 2018 versus post-intervention period: October 2018 until December 2020. Data are in n (%). NA: not available. [x] Fisher’s exact test. *Alpha<0.05

	SGA (n=191)			Non-SGA (n=510)		
Mode of delivery	Pre-intervention period (n=68)	Post-intervention period (n=123)	p-value	Pre-intervention period (n=160)	Post-Intervention period (n=350)	p-value
**Induction of labour**	30 (44.1)	39 (31.7)	0.087	48 (30.0)	88 (25.1)	0.250
**Vaginal delivery**	56 (82.4)	102 (82.9)	0.827	122 (76.3)	295 (84.3)	0.065
**Vacuum assisted delivery[x]**	2 (2.9)	2 (1.6)	0.617 [x]	4 (2.5)	9 (2.6)	1.000
**Caesarean section**	10 (14.7)	19 (15.4)	0.891	34 (21.3)	46 (13.1)	**0.019***
- **Primary caesarean section[x]**	5 (7.4)	12 (10.6)	0.608	23 (14.4)	32 (9.1)	0.077
- **Secondary caesarean section[x]**	5 (7.4)	7 (5.7)	0.757	11 (6.9)	14 (4.0)	0.163
Neonatal outcomes						
**Meconium-containing amniotic fluid**	10 (14.7)	15 (12.2)	0.622	21 (13.1)	48 (13.7)	0.857
**Apgar score <7 (5 minutes post-partum) [x]**	0 (0.0)	3 (2.4)	0.265	3 (1.9)	5 (1.4)	0.710
**Ph <7.05 (5 minutes post-partum) [x]**	0 (0.0)	2 (1.6)	0.539	2 (1.3)	3 (0.9)	0.651
**Paediatrician involvement**	63 (92.6)	101 (82.1)	**0.045***	60 (37.5)	124 (35.4)	0.651
**Perinatal mortality[x]**	0 (0.0)	0 (0.0)	NA	1 (0.6)	1 (0.3)	0.529

Discussion

The implementation of the care pathway for pregnant women with a history of SGA led to a reduction in prenatal secondary care consultations, and fewer labour inductions. Additionally, the number of births in primary care increased, with no significant adverse impact on neonatal outcomes in the post-intervention period compared to the pre-intervention period.

Our finding regarding the decline in mean number of antenatal secondary care consultations might be interpreted as an indicator of improved efficacy of care (18). Furthermore, previous literature found that reduced antenatal consultations are associated with fewer interventions and improvement of perinatal outcomes in low-risk pregnancies (19). Another randomized controlled trial in a tertiary academic centre found that a reduced frequency prenatal care model with fewer onsite appointments supplemented with virtual visit was associated with higher patient satisfaction without compromising perinatal outcomes (20)

After implementation of the care pathway, there were fewer inductions of labour (IOL) compared to the pre-intervention period. Unfortunately, the reason for IOL were not available for analysis within our study. This finding is not in line with previous research that found no association between model of maternity care and IOL rate (12)

Our study demonstrates that following the implementation of the SGA care pathway, more women gave birth in a primary care setting with similar perinatal outcomes. This shift toward primary care is significant, as it allowed more women with intermediate-risk pregnancies to deliver in primary care compared to the period before the integrated care pathway was implemented. Providing care in a primary care setting has been shown to positively impact patient experience and perinatal outcomes. (12). Similarly, shifting care to primary maternity care settings has proven feasible for women at risk of preterm birth.(20)

Regarding our secondary objectives, stratified by whether a woman delivered an SGA neonate or a neonate with normal birthweight, we found that following implementation of the

SGA care pathway, the number of caesarean sections decreased in women who, in their current pregnancy, gave birth to an infant with a normal birthweight (referred to as non-SGA group). We hypothesize the integrated care pathway with a clear structure probably has facilitated this decline, although proof of causality is difficult with this 'before-after' study design. The integrated patient record enabled enhanced peer consultation between primary and secondary care providers. Together with the structured care pathway, this led to improved informational continuity of care and patient-centred care, leveraging knowledge of prior events and individual circumstances. (21,22) This finding aligns with a systematic review showing that organisational interventions, such as team midwifery models, are associated with reduced caesarean section rates.(23)

We observed a 10% decrease in paediatric involvement in the SGA group, to 82.1% following implementation of the integrated SGA care pathway. Assessment of all 101 cases separately revealed that paediatric involvement was often omitted after uncomplicated home births with a birthweight just below the 10th percentile. In these cases, feeding advice was provided by the attending community midwife, and hospital transfer was deemed unnecessary. No adverse neonatal outcomes, such as neonatal admissions, were reported in these cases. This suggests that post-homebirth hospital transfers for glucose monitoring may pose a barrier in certain situations.

Strengths and limitations

A key strength of this study is its provision of insights into the implementation of an integrated care pathway in clinical practice, encompassing all pregnant women in the region. From a value-based healthcare perspective, we evaluated this integrated care pathway to identify areas for future improvement. However, its retrospective design is a limitation, and we were not able to include long-term neonatal outcomes. Finally, the concurrent COVID-19 pandemic may have contributed to the observed reduction in antenatal consultations in secondary care in the end of 2020.

Future research should focus on a comprehensive cost-effectiveness analysis to provide robust evidence regarding the implementation of integrated maternity care. While this study has assessed the integrated care pathway from a value-based healthcare perspective and assessed the care process, which provide insights into care efficacy, a more comprehensive economic evaluation is required to draw more robust conclusions.

Conclusion

The implementation of the care pathway for pregnant women with a history of SGA resulted in a reduction in antenatal secondary care consultations and fewer inductions of labour. Additionally, the number of births in primary care increased, with no significant adverse impact on neonatal outcomes in the post-intervention period compared to the pre-intervention period.

Financial Disclosure Statement

ZonMw 10100022020013. The funders had no role in study design, data collection and analysis, decision to publish, or preparation of the manuscript.

Ethics statement

The data used in this study were provided by the regional hospital, and the Netherlands Perinatal Registry , and the project leader of the study, who is one of the co-authors, was involved in the development of the database. The anonymisation of the data was performed prior to analysis and in accordance with institutional protocols. The retrospective use of anonymized medical records exempted institutional review of the Medical Ethics Committee. This complies with national guidelines (https://english.ccmo.nl/).

Acknowledgements

The Netherlands Perinatal Registry, Utrecht, the Netherlands, kindly provided permission for the data analyses included.

References

1. Stuurgroep zwangerschap en geboorte. Veilige zorg rond zwangerschap en geboorte. 2009;1–86.

2. Kerncijfers middelengebruik van vrouwen rond de zwangerschap in 2021.

3. Broeders L, Achterberg PW, Waelput AJM, Ravelli ACJ, Kwee A, Groenendaal F, et al. [Decrease in foetal and neonatal mortality in the Netherlands; comparison with other Euro-Peristat countries in 2004, 2010 and 2015]. Ned Tijdschr Geneeskd. 2019 Jul;163.

4. EUROPEAN PERINATAL HEALTH REPORT. [cited 2022 Jul 14]; Available from: www.europeristat.com

5. Bonsel GJ (Gouke), Birnie E (Erwin), Denktaş S (Semiha), Steegers EAP (Eric), Poeran VJJ (Jashvant). Lijnen in de Perinatale Sterfte [Internet]. 2010. Available from: http://www.narcis.nl/publication/RecordID/oai:repub.eur.nl:23454

6. Katz J, Lee ACC, Kozuki N, Lawn JE, Cousens S, Blencowe H, et al. Mortality risk in preterm and small-for-gestational-age infants in low-income and middle-income countries: a pooled country analysis. Lancet (London, England) [Internet]. 2013 [cited 2023 Feb 22];382(9890):417–25. Available from: https://pubmed.ncbi.nlm.nih.gov/23746775/

7. Lee ACC, Katz J, Blencowe H, Cousens S, Kozuki N, Vogel JP, et al. National and regional estimates of term and preterm babies born small for gestational age in 138 low-income and middle-income countries in 2010. Lancet Glob Heal [Internet]. 2013 Sep [cited 2023 Feb 22];1(1). Available from: https://pubmed.ncbi.nlm.nih.gov/25103583/

8. Pulver LS, Guest-Warnick G, Stoddard GJ, Byington CL, Young PC. Weight for gestational age affects the mortality of late preterm infants. Pediatrics [Internet]. 2009

Jun [cited 2023 Feb 22];123(6). Available from: https://pubmed.ncbi.nlm.nih.gov/19482740/

9. Martin E, Ayoub B, Miller YD. A systematic review of the cost-effectiveness of maternity models of care. BMC Pregnancy Childbirth [Internet]. 2023 Dec 1 [cited 2025 Jan 4];23(1):859. Available from: https://pmc.ncbi.nlm.nih.gov/articles/PMC10717830/

10. Kroll-Desrosiers AR, Crawford SL, Moore Simas TA, Rosen AK, Mattocks KM. Improving Pregnancy Outcomes through Maternity Care Coordination: A Systematic Review. Women's Heal Issues [Internet]. 2016;26(1):87–99. Available from: http://dx.doi.org/10.1016/j.whi.2015.10.003

11. Sandall J, Fernandez Turienzo C, Devane D, Soltani H, Gillespie P, Gates S, et al. Midwife continuity of care models versus other models of care for childbearing women. Cochrane Database Syst Rev. 2024 Apr 10;2024(4).

12. Chapman S. Midwife-led continuity models versus other models of care for childbearing women, Sandall et al (2015). Vol. 19, Practising Midwife. 2016. 34–35 p.

13. Hermans A, Spaan J, Hermus M, Visser J, Research Collaboration A, Franx A, et al. Implementation of integrated maternity care in the southwestern region of the Netherlands: evaluation of its effect on preterm birth, low birthweight infants and number of secondary care consultations. BMJ Open [Internet]. 2024 [cited 2024 Oct 29];14:69556. Available from: http://bmjopen.bmj.com/

14. Donnellan-Fernandez RE, Creedy DK, Callander EJ. Cost-effectiveness of continuity of midwifery care for women with complex pregnancy: A structured review of the literature. Health Econ Rev. 2018;8(1).

15. Yeshitila YG, Gold L, Abimanyi-Ochom J, Riggs E, Daba TT, Le HND. Effectiveness and cost-effectiveness of models of maternity care for women from migrant and

refugee backgrounds in high-income countries: A systematic review. Soc Sci Med. 2024 Oct 1;358:117250.

16. Hoftiezer L, Hof MHP, Dijs-Elsinga J, Hogeveen M, Hukkelhoven CWPM, van Lingen RA. From population reference to national standard: new and improved birthweight charts. Am J Obstet Gynecol [Internet]. 2019 Apr 1 [cited 2023 Feb 22];220(4):383.e1-383.e17. Available from: http://www.ajog.org/article/S0002937818322336/fulltext

17. Perinatale sterfte | CBS [Internet]. [cited 2025 Jan 23]. Available from: https://www.cbs.nl/nl-nl/onze-diensten/methoden/begrippen/perinatale-sterfte

18. Peahl AF, Gourevitch RA, Luo EM, Fryer KE, Moniz MH, Dalton VK, et al. Right-Sizing Prenatal Care to Meet Patients' Needs and Improve Maternity Care Value. Obstet Gynecol. 2020;135(5):1027–37.

19. Carter EB, Tuuli MG, Caughey AB, Odibo AO, Macones GA, Cahill AG. Number of prenatal visits and pregnancy outcomes in low-risk women. J Perinatol. 2016 Mar 1;36(3):178–81.

20. Butler Tobah YS, LeBlanc A, Branda ME, Inselman JW, Morris MA, Ridgeway JL, et al. Randomized comparison of a reduced-visit prenatal care model enhanced with remote monitoring. Am J Obstet Gynecol [Internet]. 2019 Dec 1 [cited 2025 Jan 27];221(6):638.e1-638.e8. Available from: http://www.ajog.org/article/S0002937819308105/fulltext

21. Haggerty JL, Reid RJ, Freeman GK, Starfield BH, Adair CE, McKendry R. Continuity of care: a multidisciplinary review. BMJ Br Med J [Internet]. 2003 Nov 11 [cited 2023 Feb 28];327(7425):1219. Available from: /pmc/articles/PMC274066/

22. Vos JFJ, Boonstra A, Kooistra A, Seelen M, Van Offenbeek M. The influence of electronic health record use on collaboration among medical specialties. BMC Health Serv Res. 2020;20(1):1–11.

23. Chapman A, Nagle C, Bick D, Lindberg R, Kent B, Calache J, et al. Maternity service organisational interventions that aim to reduce caesarean section: a systematic review and meta-analyses. [cited 2023 Nov 5]; Available from: https://doi.org/10.1186/s12884-019-2351-2

Anne Hermans
Department of Gynaecology and Obstetrics
Erasmus Medical Center, Rotterdam
the Netherlands

Sluis, 20 September 2025

Dear Editor,
Attached, please find our manuscript entitled "**Introducing an integrated maternity care pathway for women with a history of small-for-gestational-age: evaluation of its effect on care process and clinical outcomes"** which we would like to be considered for publication in *PLOS One* medical journal.

We would like to thank the reviewers for their time and valuable comments. We have carefully considered all points raised and have revised and improved the manuscript accordingly.
Thank you for considering our paper for publication in your journal.

We believe that our study results may be of interest to a wide audience representing *PLOS One* medical journal readers that include clinicians involved in maternity care. Please do not hesitate to reach out if you have any questions.

Yours sincerely (on behalf of all authors),

Anne Hermans, MD

Please include the following items when submitting your revised manuscript:

- A rebuttal letter that responds to each point raised by the academic editor and reviewer(s). You should upload this letter as a separate file labeled 'Response to Reviewers'.
- A marked-up copy of your manuscript that highlights changes made to the original version. You should upload this as a separate file labeled 'Revised Manuscript with Track Changes'.
- An unmarked version of your revised paper without tracked changes. You should upload this as a separate file labeled 'Manuscript'.

Journal Requirements:

When submitting your revision, we need you to address these additional requirements.

1. Please ensure that your manuscript meets PLOS ONE's style requirements, including those for file naming. The PLOS ONE style templates can be found at

https://journals.plos.org/plosone/s/file?id=wjVg/PLOSOne_formatting_sample_main_body.pdf and

https://journals.plos.org/plosone/s/file?id=ba62/PLOSOne_formatting_sample_title_authors_affiliations.pdf

2. We note that you have indicated that there are restrictions to data sharing for this study. For studies involving human research participant data or other sensitive data, we encourage authors to share de-identified or anonymized data. However, when data cannot be publicly shared for ethical reasons, we allow authors to make their data sets available upon request. For information on unacceptable data access restrictions, please see http://journals.plos.org/plosone/s/data-availability#loc-unacceptable-data-access-restrictions.

Before we proceed with your manuscript, please address the following prompts:

a) If there are ethical or legal restrictions on sharing a de-identified data set, please explain them in detail (e.g., data contain potentially identifying or sensitive patient information, data are owned by a third-party organization, etc.) and who has imposed them (e.g., a Research Ethics Committee or Institutional Review Board, etc.). Please also provide contact information for a data access committee, ethics committee, or other institutional body to which data requests may be sent.

b) If there are no restrictions, please upload the minimal anonymized data set necessary to replicate your study findings to a stable, public repository and provide us with the relevant URLs, DOIs, or accession numbers. Please see http://www.bmj.com/content/340/bmj.c181.long for guidelines on how to de-identify and prepare clinical data for publication. For a list of recommended repositories, please see https://journals.plos.org/plosone/s/recommended-repositories. You also have the option of uploading the data as Supporting Information files, but we would recommend depositing data directly to a data repository if possible.

Please update your Data Availability statement in the submission form accordingly.

We thank the Editor for the opportunity to clarify the data sharing and ethical considerations related to our study. We would like to confirm that all data used for analysis were pseudo anonymised prior to use, in accordance with institutional protocols and national ethical standards. The data were provided by the regional hospital and the Netherlands Perinatal Registry. One of the co-authors, who serves as the project leader, was involved in the development and preparation of the database. As the study involved retrospective analysis of pseudo anonymised medical records, it was exempt from

institutional ethical review, in line with national regulations as outlined by the Central Committee on Research Involving Human Subjects (CCMO) in the Netherlands (https://english.ccmo.nl). We further confirm that the dataset is complete and that there are no restrictions on data sharing. All data relevant to the findings reported in the manuscript are available. The pseudo anonymised dataset can be made available upon reasonable request to the corresponding author, in full accordance with the journal's data sharing policy. Should the Editor or reviewers require any further information or clarification, we would be more than happy to provide additional details as needed.

3. We note that your Data Availability Statement is currently as follows: All relevant data are within the manuscript and its Supporting Information files.

Please confirm at this time whether or not your submission contains all raw data required to replicate the results of your study. Authors must share the "minimal data set" for their submission. PLOS defines the minimal data set to consist of the data required to replicate all study findings reported in the article, as well as related metadata and methods (https://journals.plos.org/plosone/s/data-availability#loc-minimal-data-set-definition).

We confirm that our submission contains all raw data required to replicate the results of our study. All relevant data, including the data required to replicate all study findings, as well as related metadata and methods), are provided within the manuscript and its Supporting Information files.

The data were pseudo anonymised prior to use, in accordance with institutional protocols and national ethical standards. The data were provided by the regional hospital and the Netherlands Perinatal Registry. One of the co-authors, who serves as the project leader, was involved in the development and preparation of the database. As the study involved retrospective analysis of pseudo anonymised medical records, it was exempt from institutional ethical review, in line with national regulations as outlined by the Central Committee on Research Involving Human Subjects (CCMO) in the Netherlands (https://english.ccmo.nl). We further confirm that the dataset is complete and that there are no restrictions on data sharing. All data relevant to the findings reported in the manuscript are available. The pseudo anonymised dataset can be made available upon reasonable request to the corresponding author, in full accordance with the journal's data sharing policy. Should the Editor or reviewers require any further information or clarification, we would be more than happy to provide additional details as needed.

For example, authors should submit the following data:

- The values behind the means, standard deviations and other measures reported;
- The values used to build graphs;
- The points extracted from images for analysis.

Authors do not need to submit their entire data set if only a portion of the data was used in the reported study.

If your submission does not contain these data, please either upload them as Supporting Information files or deposit them to a stable, public repository and provide us with the relevant URLs, DOIs, or accession numbers. For a list of recommended repositories, please see https://journals.plos.org/plosone/s/recommended-repositories.

If there are ethical or legal restrictions on sharing a de-identified data set, please explain them in detail (e.g., data contain potentially sensitive information, data are owned by a third-party organization, etc.) and who has imposed them (e.g., an ethics committee). Please also provide

contact information for a data access committee, ethics committee, or other institutional body to which data requests may be sent. If data are owned by a third party, please indicate how others may request data access.

Ethics statement

The data used in this study were provided by the regional hospital, and the Netherlands Perinatal Registry , and the project leader of the study, who is one of the co-authors, was involved in the development of the database. The anonymisation of the data was performed prior to analysis and in accordance with institutional protocols. The retrospective use of anonymized medical records exempted institutional review of the Medical Ethics Committee. This complies with national guidelines (https://english.ccmo.nl/).

Additional Editor Comments:

1. Please provide parity, the rate of hypertensive disorder in pregnancy and overt diabetes which are the main risk factors for SGA. The rate of gestational Diabetes should be also analyzed. Other risk factors for SGA should be provided in this study.

We thank the reviewer for this valuable remark. In response, we have added the requested variables including the rate of hypertensive disorders in pregnancy, overt diabetes, gestational diabetes, and other relevant risk factors for SGA to the baseline characteristics table 1. Parity was not included. We believe that these additions provide a more comprehensive overview of the baseline characteristics and strengthen the contextual interpretation of our findings.

2. The indication for aspirin use in SGA needs to be stated. Aspirin use is helpful in SGA but the guideline as an indication is not established yet.

We appreciate the editor's valuable comment on this point, as recent research on the indication for aspirin in pregnancy has not yet reached full consensus. In cases of a previous pregnancy complicated by the birth of a small-for-gestational-age (SGA) infant, low-dose aspirin is recommended in subsequent pregnancies due to the increased risk of placental insufficiency and related adverse outcomes. Specifically, several international guidelines advise the use of low-dose aspirin (typically 80–150 mg daily) in women with a history of SGA, particularly when the birthweight was below the 5th percentile. (1–4)

Treatment is most effective when initiated before 16 weeks of gestation, ideally before 12 weeks, and is usually continued until 36 weeks of gestation. The preventive effect of aspirin is attributed to its antiplatelet action, which may improve placental perfusion and reduce the recurrence risk of SGA or preeclampsia.

In our region, the recommendation is based on the Dutch Society of Obstetrics and Gynaecology (NVOG) guideline on foetal growth restriction.

We added the following text to the Methods section, lines 117-120:

As shown in Fig 1, the SGA care pathway includes antenatal counselling on the use of low-dose aspirin, in accordance with the Dutch Society of Obstetrics and Gynaecology (NVOG) guideline on fetal growth restriction. In addition, the pathway incorporates prenatal ultrasounds at 27, 31, and 35 weeks of gestation to monitor foetal growth.

2. In order to understand the circumstance in Netherlands and be generalizable to international readers, usual antenatal practice in Dutch needs to be stated.

We thank the editor for this valuable remark. To address this, we have added a brief explanation of usual antenatal practice in the Netherlands to provide context and improve generalizability for international readers. This addition can be found in the revised manuscript in the following section:

Introduction, lines 50-61.
The Netherlands employs a tiered maternity care system based on estimated risk for adverse outcomes. Primary care for low-risk pregnancies is provided by community midwives, hospital-based obstetricians manage intermediate-risk pregnancies in secondary care, and university medical centres provide tertiary care for high-risk pregnancies. Approximately 80% of pregnant women receive prenatal care across these different tiers. However, referrals from primary to secondary care can disrupt continuity, increasing the risk of losing critical medical information. Inadequate integration between these levels of care may result in duplicated procedures—such as unnecessary ultrasound examinations—thereby contributing to over-medicalisation. Therefore, The Dutch Steering Group Pregnancy and Childbirth, installed by the Ministry of Health as a response to the performance in the EURO-PERISTAT benchmark in 2008, emphasized the need for an integrated maternity care model to enhance interprofessional collaboration between primary and secondary care. (1)

Introduction, lines 74-82
In the Southwestern region, these findings eventually led to the foundation of an integrated maternity care organisation (IMCO). Within the IMCO, obstetric professionals from primary and secondary care work within an integrated patient record. (13) Furthermore, they collaboratively developed standardized protocols and pathways to enhance patient-centred care. These care pathways outline antenatal consultations and clearly set the roles of community midwives, clinical midwives, and obstetricians. Within this model, intermediate-risk pregnancies, such as those involving a history of small-for-gestational-age (SGA) infants, are managed through integrated care, with close interprofessional collaboration between primary and secondary care providers.

2. The overlap between pre and post intervention needs explanation as the reviewer recommended.

As of January 2018, women with a history of SGA were enrolled in the newly developed integrated SGA care pathway at the intake of a subsequent pregnancy. Prior to this, care for these women had not yet been formally structured within a standardized integrated care protocol. Consequently, the pre-intervention period was defined as women who delivered between January 2017 and September 2018, while the post-intervention period comprised women who delivered between October 2018 and December 2020. In the post-intervention group, we included women who gave birth from October 2018 until December 2020. Consequently, there is no overlap between the two groups. We added this explanation to the method section:

Methods section, lines 97-104
As of January 2018, women with a history of SGA were enrolled in the newly developed integrated SGA care pathway at the intake of a subsequent pregnancy. Prior to this, care for these women had not yet been formally structured within a standardized integrated care protocol. Consequently, the pre-intervention period was defined as women who delivered between January 2017 and September 2018, while the post-intervention period comprised women who delivered between October 2018 and December 2020. In the post-intervention group, we included women who gave birth from October 2018 until December 2020.

Reviewers' comments:

Reviewer's Responses to Questions

Comments to the Author

1. Is the manuscript technically sound, and do the data support the conclusions?

The manuscript must describe a technically sound piece of scientific research with data that supports the conclusions. Experiments must have been conducted rigorously, with appropriate controls, replication, and sample sizes. The conclusions must be drawn appropriately based on the data presented.

Reviewer #1: Yes

Reviewer #2: Yes

---

2. Has the statistical analysis been performed appropriately and rigorously?

Reviewer #1: Yes

Reviewer #2: Yes

---

3. Have the authors made all data underlying the findings in their manuscript fully available?

The PLOS Data policy requires authors to make all data underlying the findings described in their manuscript fully available without restriction, with rare exception (please refer to the Data Availability Statement in the manuscript PDF file). The data should be provided as part of the manuscript or its supporting information, or deposited to a public repository. For example, in addition to summary statistics, the data points behind means, medians and variance measures should be available. If there are restrictions on publicly sharing data—e.g. participant privacy or use of data from a third party—those must be specified.

Reviewer #1: No

Reviewer #2: Yes

We confirm that our submission contains all raw data required to replicate the results of our study. All relevant data, including the data required to replicate all study findings, as well as related metadata and methods), are provided within the manuscript and its Supporting Information files.

The data were pseudo anonymised prior to use, in accordance with institutional protocols and national ethical standards. The data were provided by the regional hospital and the Netherlands Perinatal Registry. One of the co-authors, who serves as the project leader, was involved in the development and preparation of the database. As the study involved retrospective analysis of pseudo anonymised medical records, it was exempt from institutional ethical review, in line with national regulations as outlined by the Central Committee on Research Involving Human Subjects (CCMO) in the Netherlands (https://english.ccmo.nl). We further confirm that the dataset is complete and that there are no restrictions on data sharing. All data relevant to the findings reported in the manuscript are available. The pseudo anonymised dataset can be made available upon reasonable request to the corresponding author, in full accordance with the journal's data sharing policy. Should the Editor or

reviewers require any further information or clarification, we would be more than happy to provide additional details as needed.

---

4. Is the manuscript presented in an intelligible fashion and written in standard English?

PLOS ONE does not copyedit accepted manuscripts, so the language in submitted articles must be clear, correct, and unambiguous. Any typographical or grammatical errors should be corrected at revision, so please note any specific errors here.

Reviewer #1: Yes

Reviewer #2: Yes

---

5. Review Comments to the Author

Please use the space provided to explain your answers to the questions above. You may also include additional comments for the author, including concerns about dual publication, research ethics, or publication ethics. (Please upload your review as an attachment if it exceeds 20,000 characters)

Reviewer #1: There is an overlap of pre-intervention and post-intervention time. As per the paper "The SGA care pathway was initially developed in December 2017, with clinical implementation occurring gradually between January and May 2018. In the pre intervention group, included women who gave birth between January 2017 and September
99 2018. In the post-intervention group, we included women who gave birth from 2018 until December
100 2020." I dont understand the reason for this overlap and why couldnt it be all together avoided?

As of January 2018, women with a history of SGA were enrolled in the newly developed integrated SGA care pathway at the intake of a subsequent pregnancy. Prior to this, care for these women had not yet been formally structured within a standardized integrated care protocol. Consequently, the pre-intervention period was defined as women who delivered between January 2017 and September 2018, while the post-intervention period comprised women who delivered between October 2018 and December 2020. In the post-intervention group, we included women who gave birth from October 2018 until December 2020. Consequently, there is no overlap between the two groups. We added this explanation to the method section:

Methods section, lines 97-104

As of January 2018, women with a history of SGA were enrolled in the newly developed integrated SGA care pathway at the intake of a subsequent pregnancy. Prior to this, care for these women had not yet been formally structured within a standardized integrated care protocol. Consequently, the pre-intervention period was defined as women who delivered between January 2017 and September 2018, while the post-intervention period comprised women who delivered between October 2018 and December 2020. In the post-intervention group, we included women who gave birth from October 2018 until December 2020.

One of the aim of the study was to see a reduction in the number of in-hospital visits? Does it mean inpatient visits?? thats quite confusing

We thank the reviewer for this observation and would like to clarify that we indeed assessed secondary care consultations as a measure of care efficacy.(5) By secondary care, we refer to consultations carried out by either an obstetrician or a clinical midwife in the hospital outpatient clinic.

To provide context for international readers, we have described the Dutch maternity care system and recent regional developments in care integration. The Netherlands employs a tiered maternity care system based on estimated risk for adverse outcomes: primary care for low-risk pregnancies is provided by community midwives; hospital-based obstetricians manage intermediate-risk pregnancies in secondary care; and university medical centres provide tertiary care for high-risk pregnancies. Approximately 80% of pregnant women receive prenatal care across these tiers, but referrals from primary to secondary care can disrupt continuity, risking loss of essential medical information. Poor integration between tiers may lead to redundant procedures, such as unnecessary ultrasound investigations, potentially resulting in over-medicalisation.

Our underlying hypothesis is that more integrated care, with improved cross-tier collaboration, will reduce unnecessary duplication of services and avoid non-essential consultations. Within an integrated care pathway, responsibilities and tasks of primary and secondary care providers are more clearly aligned, which facilitates efficient care allocation, prevents overlap, and promotes optimal use of healthcare resources.

Reviewer #2: Subject: REVIEWERS' COMMENT ON MANUSCRIPT NUMBER: PONE-D-25-11450

Title: Introducing an integrated maternity care pathway for women with a history of small-for-gestational-age: evaluation of its effect on care process and clinical outcomes

I would like to congratulate the authors on the manuscript submitted for review and publication, which focuses on an important aspect of maternal and perinatal care, focused on task shifting with optimal maternal-fetal outcome.
The article, the implementation of an integrated maternity care pathway for women with a history of small-for-gestational-age (SGA) pregnancies, evaluated the care pre and postdelivery, reducing the focus of care on the secondary health facility, except if required, to a integrated maternity care pathway, with deliveries within the primary level of care, assessing efficacy of the pathway by reduced antenatal in-hospital visits and labor inductions, and compared clinical outcomes before and after its implementation.
The outcomes were: a reduction in antenatal visits, fewer labour inductions, an increase in Primary Care births with standard monitoring of Neonatal Outcomes, reduced caesarean deliveries and improved neonatal outcomes.
The research article satisfies the following criteria:
1. The study presents the results of original research. Yes, it does.
2. Results reported have not been published elsewhere.
3. The experiments/ methods, statistics, and other analyses are performed to a high technical standard and are described in sufficient detail.
4. Conclusions are presented in an appropriate fashion and are supported by the data. Yes, this has been presented in an appropriate fashion, and it is supported by the data.
5. The article is presented in an intelligible fashion with good flow and is written in standard English.
6. The research meets all applicable standards for the ethics of experimentation and research integrity. Yes, they did not require ethical clearance, but they ensured deidentified data was used and ensured institutional protocol and national guidelines.
7. The article adheres to appropriate reporting guidelines and community standards for data

availability. Yes, this was done, and a statement on how to access data was included.

I recommend that this article be published as it meets high standards and has researched an area of importance in the management of women and their fetuses. It has provided insights into the implementation of an integrated care pathway in clinical practice, focusing on pregnant women with a history of SGA.

Thank you for the opportunity to review this manuscript for publication

We are grateful to the reviewer for their thoughtful feedback and the considerable time and effort they invested in evaluating our manuscript.

---

6. PLOS authors have the option to publish the peer review history of their article (what does this mean?). If published, this will include your full peer review and any attached files.

If you choose "no", your identity will remain anonymous but your review may still be made public.

Do you want your identity to be public for this peer review? For information about this choice, including consent withdrawal, please see our Privacy Policy.

Reviewer #1: **Yes:** zaheena shamsul islam

Reviewer #2: No

[NOTE: If reviewer comments were submitted as an attachment file, they will be attached to this email and accessible via the submission site. Please log into your account, locate the manuscript record, and check for the action link "View Attachments". If this link does not appear, there are no attachment files.]

While revising your submission, please upload your figure files to the Preflight Analysis and Conversion Engine (PACE) digital diagnostic tool, https://pacev2.apexcovantage.com/. PACE helps ensure that figures meet PLOS requirements. To use PACE, you must first register as a user. Registration is free. Then, login and navigate to the UPLOAD tab, where you will find detailed instructions on how to use the tool. If you encounter any issues or have any questions when using PACE, please email PLOS at figures@plos.org. Please note that Supporting Information files do not need this step.

References

1. Leitich H, Egarter C, Husslein P, Kaider A, Schemper M. A meta-analysis of low dose aspirin for the prevention of intrauterine growth retardation. Br J Obstet Gynaecol [Internet]. 1997 [cited 2025 Aug 14];104(4):450–9. Available from: https://pubmed.ncbi.nlm.nih.gov/9141582/

2. Bujold E, Roberge S, Lacasse Y, Bureau M, Audibert F, Marcoux S, et al. Prevention of preeclampsia and intrauterine growth restriction with aspirin started in early pregnancy: A meta-analysis. Obstet Gynecol [Internet]. 2010 [cited 2025 Aug 14];116(2):402–14. Available from: https://pubmed.ncbi.nlm.nih.gov/20664402/

3. NVOG-richtlijn Foetale groeirestrictie (FGR).

4. Roberge S, Odibo AO, Bujold E. Aspirin for the Prevention of Preeclampsia and Intrauterine Growth Restriction. Clin Lab Med. 2016 Jun;36(2):319–29.

5. Peahl AF, Gourevitch RA, Luo EM, Fryer KE, Moniz MH, Dalton VK, et al. Right-Sizing Prenatal Care to Meet Patients' Needs and Improve Maternity Care Value. Obstet Gynecol. 2020;135(5):1027–37.
