# Supplementary material for: Introducing an integrated maternity care pathway for women with a history of small-for-gestational-age: Evaluation of its effect on care process and clinical outcomes
Source: PLoS One. 2026 Jan 28;21(1):e0341071. doi: 10.1371/journal.pone.0341071 (PMC12851471; doi:10.1371/journal.pone.0341071)
Supplement: Attachment — Submitted filename: 250920_response to reviewers.docx [file pone.0341071.s005.docx]

Anne Hermans
Department of Gynaecology and Obstetrics
Erasmus Medical Center, Rotterdam

the Netherlands

Sluis, 20 September 2025

Dear Editor,

Attached, please find our manuscript entitled “**Introducing an integrated maternity care pathway for women with a history of small-for-gestational-age: evaluation of its effect on care process and clinical outcomes”** which we would like to be considered for publication in *PLOS One* medical journal*.*

We would like to thank the reviewers for their time and valuable comments. We have carefully considered all points raised and have revised and improved the manuscript accordingly.

Thank you for considering our paper for publication in your journal.

We believe that our study results may be of interest to a wide audience representing *PLOS One* medical journal readers that include clinicians involved in maternity care. Please do not hesitate to reach out if you have any questions.

Yours sincerely (on behalf of all authors),

Anne Hermans, MD

Please include the following items when submitting your revised manuscript:

- A rebuttal letter that responds to each point raised by the academic editor and reviewer(s). You should upload this letter as a separate file labeled 'Response to Reviewers'.
- A marked-up copy of your manuscript that highlights changes made to the original version. You should upload this as a separate file labeled 'Revised Manuscript with Track Changes'.
- An unmarked version of your revised paper without tracked changes. You should upload this as a separate file labeled 'Manuscript'.

Journal Requirements:

When submitting your revision, we need you to address these additional requirements.

1. Please ensure that your manuscript meets PLOS ONE's style requirements, including those for file naming. The PLOS ONE style templates can be found at

<https://journals.plos.org/plosone/s/file?id=wjVg/PLOSOne_formatting_sample_main_body.pdf> and

[https://journals.plos.org/plosone/s/file?id=ba62/PLOSOne_formatting_sample_title_authors_affiliations.pdf](https://track.editorialmanager.com/CL0/https:%2F%2Fjournals.plos.org%2Fplosone%2Fs%2Ffile%3Fid=ba62%2FPLOSOne_formatting_sample_title_authors_affiliations.pdf/1/010f0197a5c7e77c-87709834-d87f-4bbd-905e-6a4d7c9eae47-000000/ztWMQtYjPz7V_LI1g0WPcO6t3oAKsAvnmkmHApeTbjA=217)

2. We note that you have indicated that there are restrictions to data sharing for this study. For studies involving human research participant data or other sensitive data, we encourage authors to share de-identified or anonymized data. However, when data cannot be publicly shared for ethical reasons, we allow authors to make their data sets available upon request. For information on unacceptable data access restrictions, please see <http://journals.plos.org/plosone/s/data-availability#loc-unacceptable-data-access-restrictions>.

Before we proceed with your manuscript, please address the following prompts:

a) If there are ethical or legal restrictions on sharing a de-identified data set, please explain them in detail (e.g., data contain potentially identifying or sensitive patient information, data are owned by a third-party organization, etc.) and who has imposed them (e.g., a Research Ethics Committee or Institutional Review Board, etc.). Please also provide contact information for a data access committee, ethics committee, or other institutional body to which data requests may be sent.

b) If there are no restrictions, please upload the minimal anonymized data set necessary to replicate your study findings to a stable, public repository and provide us with the relevant URLs, DOIs, or accession numbers. Please see <http://www.bmj.com/content/340/bmj.c181.long> for guidelines on how to de-identify and prepare clinical data for publication. For a list of recommended repositories, please see <https://journals.plos.org/plosone/s/recommended-repositories>. You also have the option of uploading the data as Supporting Information files, but we would recommend depositing data directly to a data repository if possible.

Please update your Data Availability statement in the submission form accordingly.

We thank the Editor for the opportunity to clarify the data sharing and ethical considerations related to our study. We would like to confirm that all data used for analysis were pseudo anonymised prior to use, in accordance with institutional protocols and national ethical standards. The data were provided by the regional hospital and the Netherlands Perinatal Registry. One of the co-authors, who serves as the project leader, was involved in the development and preparation of the database. As the study involved retrospective analysis of pseudo anonymised medical records, it was exempt from institutional ethical review, in line with national regulations as outlined by the Central Committee on Research Involving Human Subjects (CCMO) in the Netherlands (<https://english.ccmo.nl>). We further confirm that the dataset is complete and that there are no restrictions on data sharing. All data relevant to the findings reported in the manuscript are available. The pseudo anonymised dataset can be made available upon reasonable request to the corresponding author, in full accordance with the journal’s data sharing policy. Should the Editor or reviewers require any further information or clarification, we would be more than happy to provide additional details as needed.

3. We note that your Data Availability Statement is currently as follows: All relevant data are within the manuscript and its Supporting Information files.

Please confirm at this time whether or not your submission contains all raw data required to replicate the results of your study. Authors must share the “minimal data set” for their submission. PLOS defines the minimal data set to consist of the data required to replicate all study findings reported in the article, as well as related metadata and methods (<https://journals.plos.org/plosone/s/data-availability#loc-minimal-data-set-definition>).

We confirm that our submission contains all raw data required to replicate the results of our study. All relevant data, including the data required to replicate all study findings, as well as related metadata and methods), are provided within the manuscript and its Supporting Information files.

The data were pseudo anonymised prior to use, in accordance with institutional protocols and national ethical standards. The data were provided by the regional hospital and the Netherlands Perinatal Registry. One of the co-authors, who serves as the project leader, was involved in the development and preparation of the database. As the study involved retrospective analysis of pseudo anonymised medical records, it was exempt from institutional ethical review, in line with national regulations as outlined by the Central Committee on Research Involving Human Subjects (CCMO) in the Netherlands (<https://english.ccmo.nl>). We further confirm that the dataset is complete and that there are no restrictions on data sharing. All data relevant to the findings reported in the manuscript are available. The pseudo anonymised dataset can be made available upon reasonable request to the corresponding author, in full accordance with the journal’s data sharing policy. Should the Editor or reviewers require any further information or clarification, we would be more than happy to provide additional details as needed.

For example, authors should submit the following data:

- The values behind the means, standard deviations and other measures reported;
- The values used to build graphs;
- The points extracted from images for analysis.

Authors do not need to submit their entire data set if only a portion of the data was used in the reported study.

If your submission does not contain these data, please either upload them as Supporting Information files or deposit them to a stable, public repository and provide us with the relevant URLs, DOIs, or accession numbers. For a list of recommended repositories, please see <https://journals.plos.org/plosone/s/recommended-repositories>.

If there are ethical or legal restrictions on sharing a de-identified data set, please explain them in detail (e.g., data contain potentially sensitive information, data are owned by a third-party organization, etc.) and who has imposed them (e.g., an ethics committee). Please also provide contact information for a data access committee, ethics committee, or other institutional body to which data requests may be sent. If data are owned by a third party, please indicate how others may request data access.

**Ethics statement**
The data used in this study were provided by the regional hospital, and the Netherlands Perinatal Registry , and the project leader of the study, who is one of the co-authors, was involved in the development of the database. The anonymisation of the data was performed prior to analysis and in accordance with institutional protocols. The retrospective use of anonymized medical records exempted institutional review of the Medical Ethics Committee. This complies with national guidelines (<https://english.ccmo.nl/>).

**Additional Editor Comments:**

1. Please provide parity, the rate of hypertensive disorder in pregnancy and overt diabetes which are the main risk factors for SGA. The rate of gestational Diabetes should be also analyzed. Other risk factors for SGA should be provided in this study.

We thank the reviewer for this valuable remark. In response, we have added the requested variables including the rate of hypertensive disorders in pregnancy, overt diabetes, gestational diabetes, and other relevant risk factors for SGA to the baseline characteristics table 1. Parity was not included. We believe that these additions provide a more comprehensive overview of the baseline characteristics and strengthen the contextual interpretation of our findings.

2. The indication for aspirin use in SGA needs to be stated. Aspirin use is helpful in SGA but the guideline as an indication is not established yet.

We appreciate the editor’s valuable comment on this point, as recent research on the indication for aspirin in pregnancy has not yet reached full consensus. In cases of a previous pregnancy complicated by the birth of a small-for-gestational-age (SGA) infant, low-dose aspirin is recommended in subsequent pregnancies due to the increased risk of placental insufficiency and related adverse outcomes. Specifically, several international guidelines advise the use of low-dose aspirin (typically 80–150 mg daily) in women with a history of SGA, particularly when the birthweight was below the 5th percentile. (1–4)

Treatment is most effective when initiated before 16 weeks of gestation, ideally before 12 weeks, and is usually continued until 36 weeks of gestation. The preventive effect of aspirin is attributed to its antiplatelet action, which may improve placental perfusion and reduce the recurrence risk of SGA or preeclampsia.

In our region, the recommendation is based on the Dutch Society of Obstetrics and Gynaecology (NVOG) guideline on foetal growth restriction.

**We added the following text to the Methods section, lines 117-120:**

As shown in Fig 1, the SGA care pathway includes antenatal counselling on the use of low-dose aspirin, in accordance with the Dutch Society of Obstetrics and Gynaecology (NVOG) guideline on fetal growth restriction. In addition, the pathway incorporates prenatal ultrasounds at 27, 31, and 35 weeks of gestation to monitor foetal growth.

2. In order to understand the circumstance in Netherlands and be generalizable to international readers, usual antenatal practice in Dutch needs to be stated.

We thank the editor for this valuable remark. To address this, we have added a brief explanation of usual antenatal practice in the Netherlands to provide context and improve generalizability for international readers. This addition can be found in the revised manuscript in the following section:

**Introduction, lines 50-61.**
The Netherlands employs a tiered maternity care system based on estimated risk for adverse outcomes. Primary care for low-risk pregnancies is provided by community midwives, hospital-based obstetricians manage intermediate-risk pregnancies in secondary care, and university medical centres provide tertiary care for high-risk pregnancies. Approximately 80% of pregnant women receive prenatal care across these different tiers. However, referrals from primary to secondary care can disrupt continuity, increasing the risk of losing critical medical information. Inadequate integration between these levels of care may result in duplicated procedures—such as unnecessary ultrasound examinations—thereby contributing to over-medicalisation. Therefore, The Dutch Steering Group Pregnancy and Childbirth, installed by the Ministery of Health as a response to the performance in the EURO-PERISTAT benchmark in 2008, emphasized the need for an integrated maternity care model to enhance interprofessional collaboration between primary and secondary care. (1)

**Introduction, lines 74-82**
In the Southwestern region, these findings eventually led to the foundation of an integrated maternity care organisation (IMCO). Within the IMCO, obstetric professionals from primary and secondary care work within an integrated patient record. (13) Furthermore, they collaboratively developed standardized protocols and pathways to enhance patient-centred care. These care pathways outline antenatal consultations and clearly set the roles of community midwives, clinical midwives, and obstetricians. Within this model, intermediate-risk pregnancies, such as those involving a history of small-for-gestational-age (SGA) infants, are managed through integrated care, with close interprofessional collaboration between primary and secondary care providers.

2. The overlap between pre and post intervention needs explanation as the reviewer recommended.

As of January 2018, women with a history of SGA were enrolled in the newly developed integrated SGA care pathway at the intake of a subsequent pregnancy. Prior to this, care for these women had not yet been formally structured within a standardized integrated care protocol. Consequently, the pre-intervention period was defined as women who delivered between January 2017 and September 2018, while the post-intervention period comprised women who delivered between October 2018 and December 2020. In the post-intervention group, we included women who gave birth from October 2018 until December 2020. Consequently, there is no overlap between the two groups. We added this explanation to the method section:

**Methods section, lines 97-104**
As of January 2018, women with a history of SGA were enrolled in the newly developed integrated SGA care pathway at the intake of a subsequent pregnancy. Prior to this, care for these women had not yet been formally structured within a standardized integrated care protocol. Consequently, the pre-intervention period was defined as women who delivered between January 2017 and September 2018, while the post-intervention period comprised women who delivered between October 2018 and December 2020. In the post-intervention group, we included women who gave birth from October 2018 until December 2020.

**Reviewers' comments:**

Reviewer's Responses to Questions

**Comments to the Author**

1. Is the manuscript technically sound, and do the data support the conclusions?

The manuscript must describe a technically sound piece of scientific research with data that supports the conclusions. Experiments must have been conducted rigorously, with appropriate controls, replication, and sample sizes. The conclusions must be drawn appropriately based on the data presented.

Reviewer #1: Yes

Reviewer #2: Yes

2. Has the statistical analysis been performed appropriately and rigorously?

Reviewer #1: Yes

Reviewer #2: Yes

3. Have the authors made all data underlying the findings in their manuscript fully available?

The [PLOS Data policy](http://track.editorialmanager.com/CL0/http:%2F%2Fwww.plosone.org%2Fstatic%2Fpolicies.action%23sharing/1/010f0197a5c7e77c-87709834-d87f-4bbd-905e-6a4d7c9eae47-000000/hZcQS486qlom_JBgpexo7jmFZvx8tVvZ89wl5V_NmmQ=217) requires authors to make all data underlying the findings described in their manuscript fully available without restriction, with rare exception (please refer to the Data Availability Statement in the manuscript PDF file). The data should be provided as part of the manuscript or its supporting information, or deposited to a public repository. For example, in addition to summary statistics, the data points behind means, medians and variance measures should be available. If there are restrictions on publicly sharing data—e.g. participant privacy or use of data from a third party—those must be specified.

Reviewer #1: No

Reviewer #2: Yes

We confirm that our submission contains all raw data required to replicate the results of our study. All relevant data, including the data required to replicate all study findings, as well as related metadata and methods), are provided within the manuscript and its Supporting Information files.

The data were pseudo anonymised prior to use, in accordance with institutional protocols and national ethical standards. The data were provided by the regional hospital and the Netherlands Perinatal Registry. One of the co-authors, who serves as the project leader, was involved in the development and preparation of the database. As the study involved retrospective analysis of pseudo anonymised medical records, it was exempt from institutional ethical review, in line with national regulations as outlined by the Central Committee on Research Involving Human Subjects (CCMO) in the Netherlands (<https://english.ccmo.nl>). We further confirm that the dataset is complete and that there are no restrictions on data sharing. All data relevant to the findings reported in the manuscript are available. The pseudo anonymised dataset can be made available upon reasonable request to the corresponding author, in full accordance with the journal’s data sharing policy. Should the Editor or reviewers require any further information or clarification, we would be more than happy to provide additional details as needed.

4. Is the manuscript presented in an intelligible fashion and written in standard English?

PLOS ONE does not copyedit accepted manuscripts, so the language in submitted articles must be clear, correct, and unambiguous. Any typographical or grammatical errors should be corrected at revision, so please note any specific errors here.

Reviewer #1: Yes

Reviewer #2: Yes

**5. Review Comments to the Author**

Please use the space provided to explain your answers to the questions above. You may also include additional comments for the author, including concerns about dual publication, research ethics, or publication ethics. (Please upload your review as an attachment if it exceeds 20,000 characters)

Reviewer #1: There is an overlap of pre-intervention and post-intervention time. As per the paper "The SGA care pathway was initially developed in December 2017, with clinical implementation occurring gradually between January and May 2018. In the pre intervention group, included women who gave birth between January 2017 and September
99 2018. In the post-intervention group, we included women who gave birth from 2018 until December
100 2020." I dont understand the reason for this overlap and why couldnt it be all together avoided?

As of January 2018, women with a history of SGA were enrolled in the newly developed integrated SGA care pathway at the intake of a subsequent pregnancy. Prior to this, care for these women had not yet been formally structured within a standardized integrated care protocol. Consequently, the pre-intervention period was defined as women who delivered between January 2017 and September 2018, while the post-intervention period comprised women who delivered between October 2018 and December 2020. In the post-intervention group, we included women who gave birth from October 2018 until December 2020. Consequently, there is no overlap between the two groups. We added this explanation to the method section:

**Methods section, lines 97-104**

As of January 2018, women with a history of SGA were enrolled in the newly developed integrated SGA care pathway at the intake of a subsequent pregnancy. Prior to this, care for these women had not yet been formally structured within a standardized integrated care protocol. Consequently, the pre-intervention period was defined as women who delivered between January 2017 and September 2018, while the post-intervention period comprised women who delivered between October 2018 and December 2020. In the post-intervention group, we included women who gave birth from October 2018 until December 2020.

One of the aim of the study was to see a reduction in the number of in-hospital visits? Does it mean inpatient visits?? thats quite confusing

We thank the reviewer for this observation and would like to clarify that we indeed assessed secondary care consultations as a measure of care efficacy.(5) By secondary care, we refer to consultations carried out by either an obstetrician or a clinical midwife in the hospital outpatient clinic.

To provide context for international readers, we have described the Dutch maternity care system and recent regional developments in care integration. The Netherlands employs a tiered maternity care system based on estimated risk for adverse outcomes: primary care for low-risk pregnancies is provided by community midwives; hospital-based obstetricians manage intermediate-risk pregnancies in secondary care; and university medical centres provide tertiary care for high-risk pregnancies. Approximately 80% of pregnant women receive prenatal care across these tiers, but referrals from primary to secondary care can disrupt continuity, risking loss of essential medical information. Poor integration between tiers may lead to redundant procedures, such as unnecessary ultrasound investigations, potentially resulting in over-medicalisation.

Our underlying hypothesis is that more integrated care, with improved cross-tier collaboration, will reduce unnecessary duplication of services and avoid non-essential consultations. Within an integrated care pathway, responsibilities and tasks of primary and secondary care providers are more clearly aligned, which facilitates efficient care allocation, prevents overlap, and promotes optimal use of healthcare resources.

**Reviewer #2: Subject: REVIEWERS' COMMENT ON MANUSCRIPT NUMBER: PONE-D-25-11450**

Title: Introducing an integrated maternity care pathway for women with a history of small-for-gestational-age: evaluation of its effect on care process and clinical outcomes

I would like to congratulate the authors on the manuscript submitted for review and publication, which focuses on an important aspect of maternal and perinatal care, focused on task shifting with optimal maternal-fetal outcome.
The article, the implementation of an integrated maternity care pathway for women with a history of small-for-gestational-age (SGA) pregnancies, evaluated the care pre and postdelivery, reducing the focus of care on the secondary health facility, except if required, to a integrated maternity care pathway, with deliveries within the primary level of care, assessing efficacy of the pathway by reduced antenatal in-hospital visits and labor inductions, and compared clinical outcomes before and after its implementation.
The outcomes were: a reduction in antenatal visits, fewer labour inductions, an increase in Primary Care births with standard monitoring of Neonatal Outcomes, reduced caesarean deliveries and improved neonatal outcomes.
The research article satisfies the following criteria:
1. The study presents the results of original research. Yes, it does.
2. Results reported have not been published elsewhere.
3. The experiments/ methods, statistics, and other analyses are performed to a high technical standard and are described in sufficient detail.
4. Conclusions are presented in an appropriate fashion and are supported by the data. Yes, this has been presented in an appropriate fashion, and it is supported by the data.
5. The article is presented in an intelligible fashion with good flow and is written in standard English.
6. The research meets all applicable standards for the ethics of experimentation and research integrity. Yes, they did not require ethical clearance, but they ensured deidentified data was used and ensured institutional protocol and national guidelines.
7. The article adheres to appropriate reporting guidelines and community standards for data availability. Yes, this was done, and a statement on how to access data was included.

I recommend that this article be published as it meets high standards and has researched an area of importance in the management of women and their fetuses. It has provided insights into the implementation of an integrated care pathway in clinical practice, focusing on pregnant women with a history of SGA.

Thank you for the opportunity to review this manuscript for publication

We are grateful to the reviewer for their thoughtful feedback and the considerable time and effort they invested in evaluating our manuscript.

6. PLOS authors have the option to publish the peer review history of their article ([what does this mean?](https://track.editorialmanager.com/CL0/https:%2F%2Fjournals.plos.org%2Fplosone%2Fs%2Feditorial-and-peer-review-process%23loc-peer-review-history/1/010f0197a5c7e77c-87709834-d87f-4bbd-905e-6a4d7c9eae47-000000/1DBZoYsqgbcYrsqVpOftwG33kvfdoj7kwCYa49u2oK0=217)). If published, this will include your full peer review and any attached files.


If you choose “no”, your identity will remain anonymous but your review may still be made public.


**Do you want your identity to be public for this peer review?** For information about this choice, including consent withdrawal, please see our [Privacy Policy](https://track.editorialmanager.com/CL0/https:%2F%2Fwww.plos.org%2Fprivacy-policy/1/010f0197a5c7e77c-87709834-d87f-4bbd-905e-6a4d7c9eae47-000000/o5wNT7dHRjgBDnVruPmKhtdD4GxoQm6HsvZBLelosFM=217).

Reviewer #1: **Yes:** zaheena shamsul islam

Reviewer #2: No

[NOTE: If reviewer comments were submitted as an attachment file, they will be attached to this email and accessible via the submission site. Please log into your account, locate the manuscript record, and check for the action link "View Attachments". If this link does not appear, there are no attachment files.]

While revising your submission, please upload your figure files to the Preflight Analysis and Conversion Engine (PACE) digital diagnostic tool, [https://pacev2.apexcovantage.com/](https://track.editorialmanager.com/CL0/https:%2F%2Fpacev2.apexcovantage.com%2F/1/010f0197a5c7e77c-87709834-d87f-4bbd-905e-6a4d7c9eae47-000000/o60d9yzTDvW51MkAU9XzRPw3D6EpZwVXZYTNegLfpfQ=217). PACE helps ensure that figures meet PLOS requirements. To use PACE, you must first register as a user. Registration is free. Then, login and navigate to the UPLOAD tab, where you will find detailed instructions on how to use the tool. If you encounter any issues or have any questions when using PACE, please email PLOS at [figures@plos.org](mailto:figures@plos.org). Please note that Supporting Information files do not need this step.

**References**

1.	Leitich H, Egarter C, Husslein P, Kaider A, Schemper M. A meta-analysis of low dose aspirin for the prevention of intrauterine growth retardation. Br J Obstet Gynaecol [Internet]. 1997 [cited 2025 Aug 14];104(4):450–9. Available from: https://pubmed.ncbi.nlm.nih.gov/9141582/

2.	Bujold E, Roberge S, Lacasse Y, Bureau M, Audibert F, Marcoux S, et al. Prevention of preeclampsia and intrauterine growth restriction with aspirin started in early pregnancy: A meta-analysis. Obstet Gynecol [Internet]. 2010 [cited 2025 Aug 14];116(2):402–14. Available from: https://pubmed.ncbi.nlm.nih.gov/20664402/

3.	NVOG-richtlijn Foetale groeirestrictie (FGR).

4.	Roberge S, Odibo AO, Bujold E. Aspirin for the Prevention of Preeclampsia and Intrauterine Growth Restriction. Clin Lab Med. 2016 Jun;36(2):319–29.

5.	Peahl AF, Gourevitch RA, Luo EM, Fryer KE, Moniz MH, Dalton VK, et al. Right-Sizing Prenatal Care to Meet Patients’ Needs and Improve Maternity Care Value. Obstet Gynecol. 2020;135(5):1027–37.
