# Supplementary material for: Introducing an integrated maternity care pathway for women with a history of small-for-gestational-age: Evaluation of its effect on care process and clinical outcomes
Source: PLoS One. 2026 Jan 28;21(1):e0341071. doi: 10.1371/journal.pone.0341071 (PMC12851471; doi:10.1371/journal.pone.0341071)
Supplement: Attachment — Submitted filename: 251230_response to reviewers.docx [file pone.0341071.s006.docx]

Anne Hermans
Department of Gynaecology and Obstetrics
Erasmus Medical Center, Rotterdam

the Netherlands

Amsterdam, 30 December 2025

Dear Editor,

Attached, please find our manuscript entitled “**Introducing an integrated maternity care pathway for women with a history of small-for-gestational-age: evaluation of its effect on care process and clinical outcomes”** which we would like to be considered for publication in *PLOS One* medical journal*.*

We would like to thank the reviewers for their time and valuable comments. We have carefully considered all points raised and have revised and improved the manuscript accordingly.

Thank you for considering our paper for publication in your journal.

We believe that our study results may be of interest to a wide audience representing *PLOS One* medical journal readers that include clinicians involved in maternity care. Please do not hesitate to reach out if you have any questions.

Yours sincerely (on behalf of all authors),

Anne Hermans, MD

Journal Requirements:

1. If the reviewer comments include a recommendation to cite specific previously published works, please review and evaluate these publications to determine whether they are relevant and should be cited. There is no requirement to cite these works unless the editor has indicated otherwise. 

2. Please review your reference list to ensure that it is complete and correct. If you have cited papers that have been retracted, please include the rationale for doing so in the manuscript text, or remove these references and replace them with relevant current references. Any changes to the reference list should be mentioned in the rebuttal letter that accompanies your revised manuscript. If you need to cite a retracted article, indicate the article’s retracted status in the References list and also include a citation and full reference for the retraction notice.

[Note: HTML markup is below. Please do not edit.]

Reviewer's Responses to Questions

**Comments to the Author**

1. If the authors have adequately addressed your comments raised in a previous round of review and you feel that this manuscript is now acceptable for publication, you may indicate that here to bypass the “Comments to the Author” section, enter your conflict of interest statement in the “Confidential to Editor” section, and submit your "Accept" recommendation.

Reviewer #3: All comments have been addressed

Reviewer #4: (No Response)

2. Is the manuscript technically sound, and do the data support the conclusions?

The manuscript must describe a technically sound piece of scientific research with data that supports the conclusions. Experiments must have been conducted rigorously, with appropriate controls, replication, and sample sizes. The conclusions must be drawn appropriately based on the data presented.

Reviewer #3: Yes

Reviewer #4: Yes

3. Has the statistical analysis been performed appropriately and rigorously?

Reviewer #3: Yes

Reviewer #4: Yes

4. Have the authors made all data underlying the findings in their manuscript fully available?

The [PLOS Data policy](http://www.plosone.org/static/policies.action#sharing) requires authors to make all data underlying the findings described in their manuscript fully available without restriction, with rare exception (please refer to the Data Availability Statement in the manuscript PDF file). The data should be provided as part of the manuscript or its supporting information, or deposited to a public repository. For example, in addition to summary statistics, the data points behind means, medians and variance measures should be available. If there are restrictions on publicly sharing data—e.g. participant privacy or use of data from a third party—those must be specified.

Reviewer #3: Yes

Reviewer #4: Yes

5. Is the manuscript presented in an intelligible fashion and written in standard English?

PLOS ONE does not copyedit accepted manuscripts, so the language in submitted articles must be clear, correct, and unambiguous. Any typographical or grammatical errors should be corrected at revision, so please note any specific errors here.

Reviewer #3: Yes

Reviewer #4: Yes

6. Review Comments to the Author

Please use the space provided to explain your answers to the questions above. You may also include additional comments for the author, including concerns about dual publication, research ethics, or publication ethics. (Please upload your review as an attachment if it exceeds 20,000 characters)

Reviewer #3: Great study - thanks for sharing with PLOS

The study highlights the importance of the implementation of an integrated care pathway.

Reviewer #4: The study is very good and recommended for the pulibication so that it will help others as well. However, there are few observations from my side attached in the manuscript.
**1. Detail about the care pathway should be mentioned, any bianess during the implementation

We thank the reviewer for this valuable comment. From the moment of implementation, all women with a history of delivering an SGA neonate were systematically enrolled in the SGA care pathway at the intake of a subsequent pregnancy. As of January 2018, these women were included in the newly developed integrated SGA care pathway. Prior to implementation, care for women with a previous SGA pregnancy was not formally structured within a standardized integrated care protocol, which resulted in variation in clinical management.**

**Accordingly, the pre‑intervention period was defined as women who delivered between January 2017 and September 2018, whereas the post‑intervention period included women who delivered between October 2018 and December 2020. This approach allowed for a clear distinction between routine care prior to pathway implementation and care provided within the integrated SGA care pathway.**

**To reduce the risk of implementation bias, all eligible women were enrolled based on obstetric history, without selective inclusion. The only exclusion applied to multiple pregnancies, as the care pathway was specifically designed for singleton pregnancies. Figure 1 illustrates the care timeline in detail, including the scheduled antenatal consultations and the distinction between primary and secondary care settings.**

**2. Methodology section needs to be simplified little bit, it sounds more complex**

**We thank the reviewer for this remark as well, and have revised the method section as follows (lines 110-180)

SGA care pathway: overview**

**Before the implementation of the SGA care pathway, there was no structured protocol for managing intermediate-risk pregnancies, leading to considerable practice variation within the IMCO. The integrated care pathway introduced a more systematic approach by clearly assigning roles to primary and secondary care providers and scheduling multidisciplinary antenatal consultations at defined intervals.**

**As shown in Fig 1, the SGA care pathway includes antenatal counselling on the use of low-dose aspirin, based on the Dutch Society of Obstetrics and Gynaecology (NVOG) guideline on foetal growth restriction. It also incorporates prenatal ultrasounds at 27, 31, and 35 weeks of gestation to monitor foetal growth. Foetal growth restriction was defined as an estimated foetal weight (EFW) or abdominal circumference (AC) below the 10th percentile, or a growth deviation of ≥20 percentiles within two weeks. If no signs of foetal growth restriction were detected at the 35-week visit and no other complications were present, further management of pregnancy and labour proceeded in primary care.**

**Outcomes**

**To evaluate the impact of the SGA care pathway, two comparisons were made:**

**1) care and perinatal outcomes were compared between the period before (January 2017 to September 2018) and after (October 2018 to December 2020) the introduction of the integrated care pathway for women with previous SGA; and (2) perinatal outcomes were compared between the pre- and post-intervention periods, stratified by whether the neonate was SGA (the “SGA group”) or had a normal birthweight (the “non-SGA group”).**

**Baseline characteristics were compared between the pre- and post-intervention periods, and included maternal age, pre-pregnancy BMI, ethnicity, smoking at pregnancy intake, and residence in a deprived neighbourhood (defined as an area with social and infrastructural disadvantages).**

**Care related factors included: mean number of antenatal consultations per pregnancy per year in primary care or secondary care for the years 2017 to 2020. Antenatal consultations in primary care were available for analysis from 2018 onward as they were registered within the integrated patient record from 2018 onward, so the analysis focused on annual averages rather than direct pre-post comparisons.**

**Additional outcomes included the place and mode of delivery, based on both onset of labour and actual delivery location (primary vs. secondary care). Additionally, perinatal outcomes included the prevalence of meconium-stained amniotic fluid, an Apgar score below 7 after 5 minutes, an umbilical artery pH below 7.05, paediatric involvement, and perinatal mortality, defined as the sum of stillbirths and early neonatal deaths within the first 7 days, which is expressed per 1,000 live births.(17) Paediatric involvement was defined as any involvement of the paediatrician, which included both paediatric consultations as neonatal admissions to the ward or the NICU.**

7. PLOS authors have the option to publish the peer review history of their article ([what does this mean?](https://journals.plos.org/plosone/s/editorial-and-peer-review-process#loc-peer-review-history)). If published, this will include your full peer review and any attached files.

If you choose “no”, your identity will remain anonymous but your review may still be made public.

**Do you want your identity to be public for this peer review?** For information about this choice, including consent withdrawal, please see our [Privacy Policy](https://www.plos.org/privacy-policy).

Reviewer #3: **Yes:** Martin Mueller, MD, PhD

Reviewer #4: **Yes:** Arpita Karki

[NOTE: If reviewer comments were submitted as an attachment file, they will be attached to this email and accessible via the submission site. Please log into your account, locate the manuscript record, and check for the action link "View Attachments". If this link does not appear, there are no attachment files.]

To ensure your figures meet our technical requirements, please review our figure guidelines: https://journals.plos.org/plosone/s/figures

You may also use PLOS’s free figure tool, NAAS, to help you prepare publication quality figures: https://journals.plos.org/plosone/s/figures#loc-tools-for-figure-preparation.

NAAS will assess whether your figures meet our technical requirements by comparing each figure against our figure specifications.
